# Global patterns of antigen receptor repertoire disruption across adaptive immune compartments in COVID-19

**DOI:** 10.1073/pnas.2201541119

**Published:** 2022-08-09

**Authors:** Magdalene Joseph, Yin Wu, Richard Dannebaum, Florian Rubelt, Iva Zlatareva, Anna Lorenc, Zhipei Gracie Du, Daniel Davies, Fernanda Kyle-Cezar, Abhishek Das, Sarah Gee, Jeffrey Seow, Carl Graham, Dilduz Telman, Clara Bermejo, Hai Lin, Hosseinali Asgharian, Adam G. Laing, Irene del Molino del Barrio, Leticia Monin, Miguel Muñoz-Ruiz, Duncan R. McKenzie, Thomas S. Hayday, Isaac Francos-Quijorna, Shraddha Kamdar, Richard Davis, Vasiliki Sofra, Florencia Cano, Efstathios Theodoridis, Lauren Martinez, Blair Merrick, Karen Bisnauthsing, Kate Brooks, Jonathan Edgeworth, John Cason, Christine Mant, Katie J. Doores, Pierre Vantourout, Khai Luong, Jan Berka, Adrian C. Hayday

**Affiliations:** ^a^Peter Gorer Department of Immunobiology, School of Immunology and Microbial Sciences, King’s College London, London, SE1 9RT, United Kingdom;; ^b^Immunosurveillance Laboratory, The Francis Crick Institute, London, NW1 1AT, United Kingdom;; ^c^Breast Cancer Now Research Unit, King’s College London, London, SE1 9RT, United Kingdom;; ^d^Department of Medical Oncology, Guy’s and St. Thomas’ NHS Foundation Trust, London, SE1 9RT, United Kingdom;; ^e^UCL Cancer Institute, University College London, London, WC1E 6DD, United Kingdom;; ^f^Roche Diagnostics Solutions, Pleasanton, CA, 94588;; ^g^Department of Plastic and Reconstructive Surgery, Royal Free NHS Foundation Trust, London, NW3 2QG, United Kingdom;; ^h^London School of Hygiene & Tropical Medicine, London, WC1E 7HT, United Kingdom;; ^i^Department of Infectious Diseases, School of Immunology & Microbial Sciences, King's College London, London, SE1 9RT, United Kingdom;; ^j^Regeneration Group, Wolfson Centre for Age-Related Diseases, Institute of Psychiatry, Psychology and Neuroscience, King’s College London, London, SE5 8AB, United Kingdom;; ^k^Research and Development Department, Guy's and St. Thomas' NHS Foundation Trust, London, SE1 7EH, United Kingdom;; ^l^Centre for Clinical Infection and Diagnostics Research, Department of Infectious Diseases, Guy’s and St Thomas’ NHS Foundation Trust, London, SE1 7EH, United Kingdom;; ^m^Infectious Diseases Biobank, Department of Infectious Diseases, School of Immunology and Microbial Sciences, King’s College London, London, SE1 9RT, United Kingdom

**Keywords:** next-generation sequencing, antigen-receptor repertoires, SARS-CoV-2, adaptive immune responses, immunoPETE

## Abstract

Whereas pathogen-specific T and B cells are a primary focus of interest during infectious disease, we have used COVID-19 to ask whether their emergence comes at a cost of broader B cell and T cell repertoire disruption. We applied a genomic DNA-based approach to concurrently study the immunoglobulin-heavy (IGH) and T cell receptor (TCR) β and δ chain loci of 95 individuals. Our approach detected anticipated repertoire focusing for the IGH repertoire, including expansions of clusters of related sequences temporally aligned with SARS-CoV-2–specific seroconversion, and enrichment of some shared SARS-CoV-2–associated sequences. No significant age-related or disease severity–related deficiencies were noted for the IGH repertoire. By contrast, whereas focusing occurred at the TCRβ and TCRδ loci, including some TCRβ sequence–sharing, disruptive repertoire narrowing was almost entirely limited to many patients aged older than 50 y. By temporarily reducing T cell diversity and by risking expansions of nonbeneficial T cells, these traits may constitute an age-related risk factor for COVID-19, including a vulnerability to new variants for which T cells may provide key protection.

## Significance

While making virus-specific immune responses to SARS-CoV-2, residual B and T cell diversity are key to making responses to concurrent co-infections and/or cancers. We applied an antigen receptor–sequencing technology and provide a comprehensive description of the global immune repertoire in hospitalized and nonhospitalized individuals infected with SARS-CoV-2. Although B cell diversity predictably narrowed, significant narrowing of αβ and γδ T cell diversity was unexpectedly observed only in those aged over 50, which is a major inflexion point for COVID-19–associated mortality. Such a discrepancy in how older persons mount T cell responses to a new virus may be considered a risk factor for the elderly, particularly vis-à-vis new virus variants against which T cell immunity may be particularly important.

Severe disease and death caused by SARS-CoV-2 infection appear to be largely due to failures and/or dysregulation of the immune response in vulnerable populations (1). Thus, most SARS-CoV-2 infections are asymptomatic or pauci-symptomatic, particularly among younger people, who, by measures of diversity and functional responses, have greater adaptive immunocompetence than do the elderly (2, 3).

In addition to providing host protection, adaptive immune functions may contribute pathologic mediators, including B cell autoreactivities associated with specific disease-related characteristics in many patients with COVID-19 (4, 5). Additionally, early B cell responses after SARS-CoV-2 infection in some donors are enriched in cross-reactive memory B cells, including those against seasonal coronaviruses, which are of uncertain protective benefit (6). Collectively, these examples illustrate the importance of considering the dynamics of immune responses beyond those that are pathogen specific.

Over 2 y since the start of the pandemic, age remains the most evident predisposing factor for COVID-19 severity (7), evoking the increased susceptibility of older persons to other newly emerged viruses, including West Nile virus and SARS-CoV-1 (8, 9). The sequence richness of the CD4^+^ and CD8^+^ T cell receptor (TCR) repertoires is reduced in older persons relative to younger persons (10) and may underlie such vulnerabilities. This raises the question of how age might affect the mobilization of antigen-receptor repertoires in response to SARS-CoV-2 infection.

The COVID-19 pandemic has provided a unique opportunity to interrogate naïve responses to a novel human pathogen. To examine this at scale, we describe here the application of immunoPETE, a newly developed, genomic DNA (gDNA)-based technique permitting simultaneous characterization of antigen-receptor repertoires for the major adaptive lymphocyte subsets: B cells (immunoglobulin heavy chain [IGH] sequences); αβ T cells (TCRβ chain and TRB sequences), which comprise two distinct subsets; CD4^+^ and CD8^+^ cells; and γδ T cells (TCRδ chain and TRD sequences), which comprise Vδ1^+^ and Vδ2^+^ T cells.

Whereas many studies have offered important insights into antigen-receptor repertoires in active COVID-19 (11–15), our study builds on those by uniquely harnessing an array of technical and methodological approaches. Thus, immunoPETE combines gDNA-based sequencing, used in other COVID-19 repertoire studies (15), with unique molecular identifier (UMI)-based deduplication of clonal expansions and correction of PCR sequencing errors; cell sorting to segregate functionally distinct CD4^+^ and CD8^+^ populations; human leukocyte antigen (HLA) typing to enhance clustering and determination of shared specificities within CD4^+^ and CD8^+^ T cell populations; and TRD sequencing permitting a real-time comparison of the responses of qualitatively distinct Vδ1 and Vδ2 subsets to a defined human virus infection.

Studying 95 individuals comprising hospital-treated patients with COVID-19 and seropositive [sero(+)] and seronegative [sero(−)] individuals, we found clear adaptive response patterns in each lymphocyte subset studied. Repertoire focusing at the IGH locus was generalizable, with the expansion of related sequence clusters temporally aligned with seroconversion. Whereas TRB repertoires also showed similar shared sequence expansions, these were conspicuously buffered by the scale and diversity of the T lymphocyte compartments in most individuals aged <50 y, whereas they exerted much more disruptive effects in many individuals aged ≥50 y, who likewise displayed major disruptions in TRD repertoire diversity. Such impacts may limit the capacity to recognize diverse challenges (e.g., coinfections and emerging new variants) and may afford undue prominence to expanding nonneutralizing and/or self-reactive clones. Thus, they should be considered an additional age-related disease risk, monitoring of which may help inform the management of COVID-19 and of other infections.

## Results

### Antigen-Receptor Repertoires in COVID-19.

**Fig. 1.**
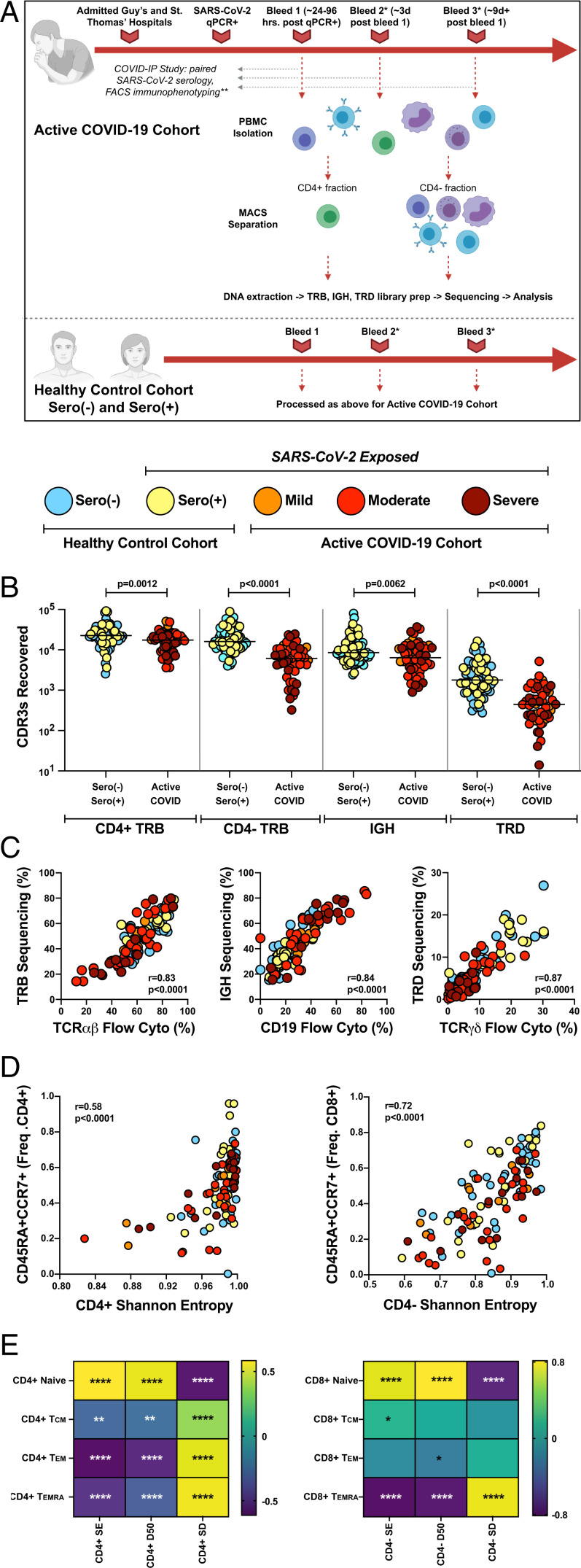
Application of immunoPETE to the COVID-IP Study cohort enables efficient, high fidelity, and quantitative recovery of TRB, IGH, and TRD CDR3s from peripheral blood mononuclear cells (PBMCs). (*A*) Summary of workflow for samples recruited into the present study. MACS: magnetic-activated cell sorting. *Not all donors had longitudinal blood sampling. **Not all healthy control samples were run through the full COVID-IP Study pipeline. All samples had SARS-CoV-2 serology data. (*B*) Recovery of CDR3s from sero(–) (*n* = 47), sero(+) (*n* = 26), mild (*n* = 10), moderate (*n* = 26), and severe (*n* = 16 CD4^+^; *n* = 15 CD4^−^ fraction) samples by immunoPETE. gDNA (2 × 250-ng replicates) was used for CD4^+^ library preparation (prep) and 1,000 ng of gDNA (4 × 250-ng replicates) was used for CD4^−^ library preparation. Bar indicates the median value. Mann–Whitney *U* test results are shown. (*C*) Correlation between immunoPETE and flow cytometry (cyto). Percentage of TRB, IGH, and TRD CDR3s recovered by immunoPETE sequencing in the CD4^−^ fraction (*y*-axis) versus percentage of CD3^+^ (*n* = 112), CD19^+^ (*n* = 112), and TCRγδ^+^ (*n* = 112) assayed by flow cytometry (*x*-axis) of the same sample. Spearman’s correlation was used for analysis. (*D*) Correlation of overall CD4^+^ TRB (*Left*) and CD4^−^ TRB (*Right*) diversity with frequency (freq) of naïve (CD45RA^+^CCR7^+^) CD4^+^ (*n* = 107) or CD8^+^ T cells (*n* = 106), respectively, as previously reported in the COVID-IP Study. Spearman’s correlation was used for analysis. Note that the color code for each cohort is maintained throughout all the figures that follow and the criteria for cohorts are detailed in the *SI Appendix*, *Materials and Methods*. (*E*) Correlation of overall CD4^+^ TRB (*Left*) and CD4^−^ TRB (*Right*) diversity metrics. Shannon entropy (SE), D50, Simpson’s dominance (SD) with frequency of naïve (CD45RA^+^CCR7^+^), T_CM_ (CD45RA^−^CCR7^+^T central memory), T_EM_ (CD45RA^−^CCR7^−^T effector memory), and T_EMRA_ (CD45RA^+^CCR7^−^T effector memory RA) CD4^+^ (*n* = 107), or CD8^+^ T cells (*n* = 106), respectively, as previously reported in the COVID-IP study. Color scale denotes Spearman’s *r*. Unadjusted *P* values are displayed. *P < 0.05, **P < 0.01, ***P < 0.001, ****P < 0.0001.

To understand the impact of SARS-CoV-2 infection on T and B cell repertoires, we applied the protocol shown in Fig. 1*A* to compare 32 hospital-treated patients with COVID-19 (active COVID-19); 20 healthy, convalescent, sero(+) individuals exposed to SARS-CoV-2 who were not hospitalized; and 43 nonexposed, healthy, sero(–) adult control individuals. For all participants (*SI Appendix*, Table S1), detailed immune profiling, serological analyses, and detailed clinical annotation (including World Health Organization severity scoring) were available based on their enrollment in the COVID immunophenotyping (COVID-IP) study (16).

Many individuals were aged ≥50 y (*SI Appendix*, Fig. S1*A* and Table S1), and because this is reportedly the most overt inflection point in the risk of COVID-19–associated death (17–19), we used age to segregate the antigen-receptor repertoire analyses, which were also segregated into those obtained up to 14 d after symptom onset (“early”), those obtained after that time (“late”), and those who were either asymptomatic sero(+) individuals in the community or asymptomatic hospitalized donors admitted for unrelated conditions (“unknown”). This temporal division is important for patients positive for COVID-19 who were aged ≥50 y to be compared with younger patients who invariably recovered earlier. Note that the color-code used for each cohort in Fig. 1 is maintained throughout all data figures. As expected, the severe COVID-19 subcohort was enriched in male patients (*SI Appendix*, Fig. S1*B*).

### gDNA-Based Repertoire Sequencing.

First, CD4^+^ T cells were purified from freshly harvested peripheral blood mononuclear cells by magnetic bead separation (Fig. 1*A*). We considered the CD4-depleted (CD4^−^) T cell fraction as primarily a de facto source of CD8^+^ αβ T cells (since blood harbors very few CD4^−^CD8^−^ αβ T cells); B cells; and γδ T cells.

We selected the IGH, TRB, and TRD loci for global assessment, because they encode greater reservoirs of repertoire diversity than do the immunoglobulin (Ig) light chain, TCRα, and TCRγ loci, which also have leakier allelic exclusion that confounds correspondence of productively rearranged gene sequences with cell numbers. Furthermore, TCRγ rearrangements are common in αβ T cells and prevent use of the γ chain to assess γδ T cell diversity in unsorted populations (20).

Antigen-receptor repertoires were assessed using immunoPETE, an assay that includes primer mixes targeting all known human IGHV, IGHJ, TRBV, TRBJ, TRDV and TRDJ genes, and provides unprecedented capability to concurrently sequence all three antigen-receptor chains from one sample. This is of particular importance in lymphopenic settings such as COVID-19 where clinical material may be very limited. The use of UMIs, which have hitherto mostly been applied to RNA-based antigen-receptor repertoire analyses, allows the numbers of individual clonal cells with an identical V-CDR3-J sequence to be quantified with greater confidence. Moreover, it allows identification of sequencing and PCR errors, which are apparent as rare variants of a consensus sequence with identical UMIs (*SI Appendix*, Fig. S2 *A* and *B*). The power of this error suppression is evidenced by the application of immunoPETE to two clonal T cell lines, HuT78 and Jurkat, with >99% of gDNA templates recovered being of a monoclonal origin for both cell lines (*SI Appendix*, Table S2).

Template lengths range from 170 to 210 nucleotides (interquartile range), thereby capturing comprehensive information on V, D, and J gene segment usage and on unique P and N nucleotide–mediated contributions to each CDR3. All nonproductive V-D-J rearrangements were excluded, as were any artifactual hybrid sequences. Real-world assay reproducibility was evidenced when we assessed technical duplicate libraries from the CD4^+^ fraction, using 250 ng of input DNA per library and quadruplicate libraries from the CD4^−^ fraction, which contains multiple cell types at lower representation. In each case, there was extremely high concordance between replicates in the percentage of each chain recovered, with mean SEs between 0.05% and 0.49% (*SI Appendix*, Fig. S3). Additionally, immunoPETE could reproducibly determine repertoire diversity, measured by Shannon entropy (a.k.a., Rényi α = 1) for which normalized values approaching 1.0 reflect diverse, polyclonal repertoires (21). Again, use of replicates showed very high concordance with mean SEs in Shannon entropy, ranging from 0.0004 to 0.0093 (*SI Appendix*, Fig. S4).

Any sequencing approach is theoretically vulnerable to amplification biases introduced during multiplex PCR, attributable to, for example, mispriming or selectively lesser priming efficiency. However, all target TRBV genes were recovered with relatively similar hierarchies in the CD4^+^ and CD4^−^ fractions (*SI Appendix*, Fig. S5 *A* and *B*) and, overall, there was good correlation for the relative representations of TRBV genes in both CD4^+^ and CD4^−^ fractions across donors (*SI Appendix*, Fig. S5 *C* and *D*), albeit less so in the CD4^−^ fraction, consistent with the greater oligoclonality (reduced entropy) of CD8^+^ versus CD4^+^ T cells (*SI Appendix*, Fig. S4). These data demonstrate the consistent performance of immunoPETE such that any primer bias does not preclude intrastudy comparisons. Nonetheless, to permit interstudy comparisons, we sought to assert the comparability of immunoPETE outputs with other independent, publicly available datasets. Indeed, the TRBV and TRBJ hierarchies identified by immunoPETE correlated significantly with published datasets of TRBV and TRBJ gene usage in adult peripheral blood determined either by other sequencing methods (22, 23) or by flow cytometry (24) (*SI Appendix*, Fig. S6 *A* and *B*). Thus, immunoPETE consistently recovered all TRBV genes and displayed no gross amplification biases when benchmarked against independent assays.

The effective practical application of immunoPETE was evident in our obtaining on average ∼23,000 productive TRB CDR3s from 500 ng (2 × 250-ng technical replicates) of DNA derived from the CD4^+^ fraction (Fig. 1*B*). The slightly lower recovery of CD4^+^ TRB CDR3s from patients with active COVID-19 likely reflects COVID-19–associated T cytopenia that was particularly severe for CD8^+^ T cells and Vδ2^+^ T cells (16, 25–27) and that was reflected in TRB and TRD showing the poorest sequence recoveries from the CD4^−^ fraction (Fig. 1*B*).

Importantly, there was a striking correlation of recovered IGH, TRB, and TRD CDR3 sequences with flow cytometric enumeration of cells in aliquots parallel to the sequenced samples (Spearman’s *r* > 0.8; *P* < 0.0001) (Fig. 1*C*). Given this robust and quantitative nature of immunoPETE, we could draw equivalence of productively rearranged CDR3s to cell counts, a frame of reference used from this point on. In total, 250 aggregate next-generation sequencing libraries, one each for CD4^+^ and CD4^−^ fractions, were constructed and analyzed, including some longitudinal samples primarily from patients with active COVID-19. Our analysis provides data derived from ∼6.5 million antigen-receptor chain sequences. In a rapid and basic biological test of this dataset, we observed highly significant correlations of Shannon entropy with the frequencies of CD45RA^+^CCR7^+^ αβ T cells that largely denote unexpanded, naïve T cells that would be expected to be highly diverse (Fig. 1*D*). Likewise, we found strong and inverse correlations of Shannon entropy with the frequencies of canonical memory T cell subsets that would be expected to be clonally expanded (Fig. 1*E*). Because different measures of population diversity can be variably affected by, for example, dominant clone sizes, we supplemented Shannon entropy measures with measures of D50 (the proportion of unique clones, ordered by dominance, accounting for 50% of all CDR3s; low values reflect clonal expansions), and of Simpson’s dominance (high values reflect clonal expansions). Again, we observed consistent, strong, and significant correlations of D50 and Simpson’s dominance with the frequencies of canonical memory T cell subsets (Fig. 1*E*).

### IGH Sequence Focusing and Clustering in COVID-19.

**Fig. 2.**
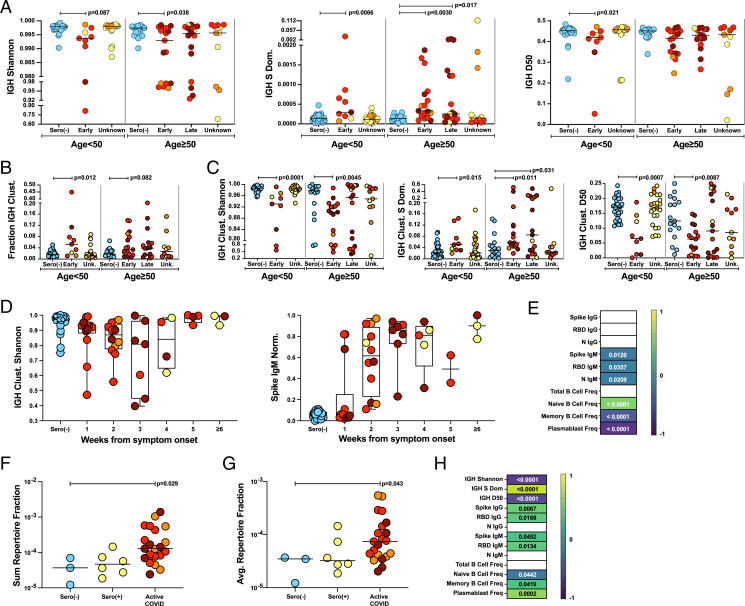
IGH clustering and repertoire focusing associated with SARS-CoV-2 exposure. (*A*) Overall IGH repertoire diversity assessed by Shannon entropy, Simpson’s dominance (S dom) and D50. The bar indicates the median value. Kruskal-Wallis test with post hoc Dunn’s test against age-matched sero(–) control individuals was used for analysis; the unadjusted *P* values are shown. (*B*) IGH CDR3s clustered (clust) as a proportion of total IGH CDR3s. The bar indicates the median. Kruskal-Wallis test with post hoc Dunn’s test against age-matched sero(–) control individuals was used for analysis; unadjusted *P* values are shown. (*C*) Within-cluster IGH repertoire diversity assessed by Shannon entropy, D50, and Simpson’s dominance. The bar indicates the median. Kruskal-Wallis test with post hoc Dunn’s test against age matched sero(–) control individuals was used for analysis; the unadjusted *P* values are shown. (*D*) IGH repertoire focusing within clusters assessed by Shannon entropy (*Left*) as well as normalized (norm) anti-spike IgM titers (*Right*) plotted by time from symptom onset (1-wk bins). (*E*) Heat map of correlations with IGH Shannon entropy in individuals with active COVID-19. Color scale denotes Spearman’s *r* with corresponding *P* values indicated within each cell. Nonsignificant correlations (*P* > 0.05) were left blank. (*F*) Fraction of IGH CDR3s occupied by the sum of CoV-AbDab IGH CDR3 matches per sample (including clustered-related sequences). The bar indicates the median. Kruskal- Wallis test with post hoc Dunn’s test against age matched sero(–) control individuals was used for analysis; the unadjusted *P* values are shown. (*G*) Average representation of CoV-AbDab IGH CDR3 matches measured by fraction of total IGH CDR3s per sample (including clustered-related sequences). The bar indicates the median. Kruskal-Wallis test with post hoc Dunn’s test against age matched sero(–) control individuals was used for analysis; the unadjusted *P* values are shown. (*H*) Heat map of correlations with the total repertoire fraction occupied by CoV-Ab Dab IGH CDR3 matches (including related clustered sequences). Color scale denotes Spearman’s *r* with corresponding *P* values indicated within each cell. Nonsignificant correlations (*P* > 0.05) were left blank. (*A*–*C*) Age: <50 y: sero(–) (*n* = 30); <50 y: early (*n* = 9); <50 y: unknown (*n* = 23); ≥50 y: sero(–) (*n* = 17); ≥50 y: early (*n* = 17); ≥50 y: late (*n* = 17); and ≥50 y: unknown (*n* = 11). (*D*) IGH Shannon entropy: sero(–) (*n* = 47); 1 wk (*n* = 14); 2 wk (*n* = 12); 3 wk (*n* = 7); 4 wk (*n* = 4); 5 wk (*n* = 4); and ≥6 wk (*n* = 3). For spike IgM: sero(–) (*n* = 34); 1 wk (*n* = 14); 2 wk (*n* = 12); 3 wk (*n* = 7); 4 wk (*n* = 5); 5 wk (*n* = 2); and ≥6 wk (*n* = 3). Freq, frequency; Unk, unknown.

Assessments of individual protection against SARS-CoV-2 are mostly based on antibody titers. Thus, with immunoPETE established as a robust method for tracking lymphocyte repertoires, we first interrogated the effect of COVID-19 on the IGH repertoire. Indeed, Shannon entropy and Simpson’s dominance measurements revealed significant IGH focusing (i.e., overrepresentation of IGH sequences suggestive of clonal expansion) for individuals sampled within 14 d of symptoms relative to sero(–) control participants (Fig. 2*A*). By D50, there was significant focusing in those aged <50 y and a trend for those aged ≥50 y (Fig. 2*A*). Donors with significant focusing spanned a spectrum of disease severities, ages, and clinical features. However, focusing had largely renormalized at later time points, suggesting a strong temporal association with disease duration (Fig. 2*A*).

It might be expected that sequence focusing following virus exposure would be reflected in expansions of structurally similar clones, with shared specificities. Using the “DefineClones.py” script in the “change-o” toolbox (*SI Appendix*, *Materials and Methods*), we found that, relative to samples from age-matched sero(–) individuals, early samples from SARS-CoV-2–exposed individuals aged <50 y showed a significant increase in the fraction of total IGH sequences that could be found in structurally related clusters. This was also true as a trend for those aged ≥50 y, and it was overt in several participants across a range of disease severities (Fig. 2*B*). The clustered fraction of the repertoire has a significantly lower Shannon entropy in SARS-CoV-2–exposed individuals than in sero(–) control participants, diagnostic of significant clonal expansions within the clusters (Fig. 2*C*). Additional metrics also revealed significantly reduced diversity of clustered IGH sequences from SARS-CoV-2–exposed individuals (Fig. 2*C*). We found a temporal drop in Shannon entropy over a time frame of ∼3 wk after symptom onset, slightly preceding but largely coincident with the development of spike-specific IgM (Fig. 2*D*).

### IGH Repertoire Architecture and Antigen-Specific Responses.

We next found that IGH focusing (low Shannon entropy) in individuals with active COVID-19 strongly correlated with high plasmablast frequency measured by flow cytometry and, albeit more modestly, with IgM antibodies to spike, RBD, and N proteins (Fig. 2*E*). To further assess whether the observed focusing was likely driven by SARS-CoV-2–reactive clones, we retrieved IGHV-CDR3-IGHJ sequences from the CoV-AbDab database (28), which contained 2,203 fully human SARS-CoV-2 reactive Igs as of July 9, 2021. Although antigen specificity reflects VH and VL sequence composition, VH chains commonly make dominant contributions (29). We found 51 instances of exact IGHV-CDR3-IGHJ matches, representing 23 unique sequences, some of which were found in multiple samples. Moreover, almost half (*n* = 11 of 23) (*SI Appendix*, Table S3) were contained within the aforementioned IGH clusters, which we showed to be expanded in SARS-CoV-2–exposed donors (Fig. 2*C*). When we included IGHV-CDR3-IGHJ sequences that were very close relatives of the direct matches, a total of 33 CoV-AbDab–associated sequences matches (*n* = 61) were found across multiple samples.

As anticipated, CoV-AbDab–associated sequences were significantly enriched in SARS-CoV-2–exposed individuals (active COVID-19, *n* = 21; sero(+), *n* = 6) relative to sero(–) individuals (*n* = 3; *P* < 0.0001 by Fisher’s exact test) (*SI Appendix*, Table S4). Moreover, those sequences were expanded within individuals with active COVID-19, relative to sero(–) individuals, since they collectively, and on average, accounted for a significantly higher fraction of an individual’s total repertoire (Fig. 2 *F* and *G*). In individuals harboring such sequences, the fraction of the repertoire to which they contributed, albeit small, was strongly correlated with repertoire focusing, with SARS-CoV-2 serology, and with plasmablast frequencies (Fig. 2*H*) consistent with SARS-CoV-2 reactive clones driving repertoire focusing, as would probably have been expected. Nonetheless, the benefit of SARS-CoV-2 reactive antibodies cannot be universally assumed. In fact, the most widely shared IGH sequence between our cohort and CoV-AbDab (CDR3-CARGFDYW) was found exclusively in SARS-CoV-2–exposed individuals (*n* = 17 of 52) but contributes to nonneutralizing S-reactive antibodies (28, 30, 31) and has been reported in Kawasaki disease (32) (see *Discussion*).

### Changes in TRB Repertoires in COVID-19.

**Fig. 3.**
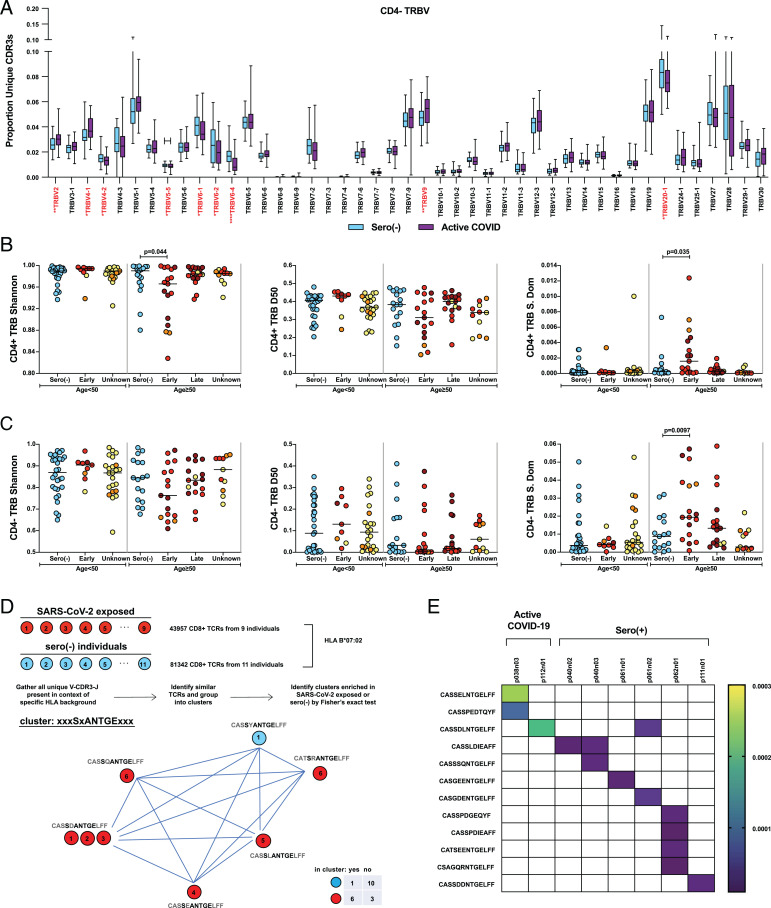
Age-related repertoire changes in CD4^+^ and CD8^+^ T cells associated with COVID-19. (*A*) Box plots of CD4^−^ TRB V gene family use as a proportion of the unique CDR3s (i.e., each unique CDR3 is treated equally regardless of clone size) in samples (*n* = 47) from sero(–) individuals and samples (*n* = 51) from individuals with active COVID-19. TRBV genes with significantly different utilization between cohorts are highlighted in red. Median, interquartile range, and range values are plotted. The Mann–Whitney U test was used for analysis; unadjusted *P* values are displayed. *P < 0.05, **P < 0.01, ***P < 0.001, ****P < 0.0001. (*B*) Overall CD4^+^ TRB repertoire diversity assessed by Shannon entropy, D50, and Simpson’s dominance (S dom). For CD4^+^ TRB metrics: age <50 y: sero(–) (*n* = 30); <50 y: early (*n* = 9); <50 y: unknown (*n* = 23); ≥50 y: sero(–) (*n* = 17); ≥50 y: early (*n* = 17); ≥50 y: late (*n* = 18); and ≥50 y: unknown (*n* = 11). (*C*) Overall CD4^−^ TRB repertoire diversity assessed by Shannon entropy, D50 and Simpson’s dominance. For CD4^−^ TRB metrics: age <50 y: sero(–) (*n* = 30); <50 y: early (*n* = 9); <50 y: unknown (*n* = 23); ≥50 y: sero(–) (*n* = 17); ≥50 y: early (*n* = 17); ≥50 y: late (*n* = 17); and ≥50 y: unknown (*n* = 11). The bar indicates the median. Kruskal-Wallis test with post hoc Dunn’s test against age-matched sero(–) control individuals; unadjusted *P* values are shown. (*D*) Defining SARS-CoV-2–associated TCR clusters. Circles represent individuals (identified by numbers) and exposure to SARS-CoV-2 (color). Clusters are built from all TCRs present in individuals with a specific HLA gene, in this example, HLA B*07:02, by grouping similar CDR3s. We tested each of such raw clusters for overrepresentation of SARS-CoV-2–exposed [active COVID-19 and sero(+)] or sero(–) individuals by Fisher’s exact test (significance set at *P* < 0.05). A representative cluster built from CD4^−^ TRB sequences of one sero(–) individual and six SARS-CoV-2–exposed individuals on an HLA B*07:02 background is shown. (*E*) Heat map showing overlap of previously described SARS-CoV-2 antigen-specific TCRs (17) with the present dataset. Shown here are 12 of 344 peptide-specific sequences on the HLA-A*02:01 background from the study by Shomuradova et al. (17). Overlap was present in two of eight individuals with active COVID-19, 4 of 9 sero(+) individuals and 0 of 17 sero(–) individuals (indicated by the shaded cell). Color scale indicates the proportion of total CD4^−^ repertoire in each sample occupied by overlapped clones.

We next turned our attention to the impact of COVID-19 on αβ T cell repertoires from the same individuals for whom IGH data had been obtained. First, we asked whether there was any evidence for viral superantigen-mediated polyclonal targeting of T cells expressing defined Vβ genes, since this has been considered to occur in COVID-19 (33) and in post–SARS-CoV-2–associated, pediatric, multisystem inflammatory syndrome (34). Our analyses failed to find significant differences between individuals with active COVID-19 and sero(–) individuals in the representation of any of *TRBV5-6*, *TRBV11-2*, *TRBV13*, *TRBV14*, or *TRBV24-1* genes that were collectively implicated in those studies (Fig. 3*A* and *SI Appendix*, Fig. S7).

Nonetheless, there were modest but significant differences in the representation of a small number of V genes among CD4^+^ and CD8^+^ T cells of individuals with active COVID-19 versus sero(–) control individuals (Fig. 3*A* and *SI Appendix*, Fig. S7). Strikingly, *TRBV6-4*, which showed the most significant difference in the CD8^+^ compartment, being underrepresented in the active COVID-19 cohort, is disproportionately expressed by “unconventional” CD8^+^ mucosal-associated invariant T cells that have been described as severely cytopenic in COVID-19 (35) (Fig. 3*A*). In sum, COVID-19 was associated with some significant Vβ-associated changes, but the overall architecture of the αβ TCR repertoire remained diverse and lacked any dominant TRBV gene representation that would reflect overt superantigen responses.

### Age-Related TCRVβ Focusing in COVID-19.

As for IGH, we next investigated how the overall architecture of the TRB repertoire was affected by COVID-19. Surprisingly, we found that by contrast to IGH, significant reductions in Shannon entropy and increases in Simpson’s dominance were not a general trait of the CD4^+^ compartment, but rather were limited to SARS-CoV-2–exposed individuals aged ≥50 y sampled within 14 d of symptom onset (Fig. 3*B*). Compared with CD4^+^ TRB, entropy values for CD8^+^ TRB were lower among sero(–) individuals (Fig. 3*C*), consistent with the greater degree to which CD8^+^ T cells show clonal expansions in response to myriad environmental exposures (36, 37). Nonetheless, the CD8^+^ TRB repertoires of SARS-CoV-2–exposed individuals aged ≥50 y sampled within 14 d of symptom onset showed a significant increase in Simpson’s dominance and a trend toward decreased Shannon entropy and D50 compared with sero(–) control individuals (Fig. 3*C*). Again, this was not a collective trait of those aged ≥50 y, as sero(–) and sero(+) (“unknown”) individuals of both age groups showed comparable diversity metrics. Thus, the differences seen in SARS-CoV-2–exposed persons ≥50 y are a function of age and of disease rather than of either alone. Suggesting similar underlying mechanisms in both αβ compartments, the degree of CD4^+^ TRBV–repertoire focusing correlated strongly with CD8^+^ TRBV–repertoire focusing. Additionally, it is evident from the color-coded data (Fig. 3 *B* and *C*) that those with the highest degree of focusing spanned a spectrum of disease severities.

To ensure that these conspicuous, age-related impacts on the αβ T cell compartment did not simply reflect lower numbers of sequences recovered (a potentially confounding variable in lymphopenia), each sample was subsampled to 1,200 (CD8^+^) or 2,400 (CD4^+^) cells (samples with fewer than these numbers of cells were excluded), and medians of metrics that were computed from 100 such resamples were reported as “subsampled” values. Strikingly, reductions in entropy and increases in dominance were now even more overt for those aged ≥50 y, being significant for CD4^+^ and CD8^+^ cells by all measures (*SI Appendix*, Fig. S8 *A* and *B*), whereas no significant changes were observed for patients with COVID-19 aged <50 y (*SI Appendix*, Fig. S8 *A* and *B*). Interestingly, by every entropy or dominance metric, the TRB repertoires of those aged ≥50 y returned toward normal at later time points after symptom onset, indicating a potential for renormalization.

Given that CD8^+^ T cells are documented to be more clonally focused than CD4^+^ T cells, there was a theoretical potential for our observations to be confounded by the purity of CD4^+^ and CD4^−^ fractions, respectively. Therefore, we examined the relationship between fraction purity and TRB clonal metrics. Evidently, the positively selected CD4^+^ fraction was very pure, with all but six samples containing <1% contamination by CD4^−^ T cells. Unsurprisingly, there was a correlation of clonal focusing with the degree of CD4^−^ contamination, but it was weak and clearly did not drive the marked clonal focusing we observed in individuals aged ≥50 y (*SI Appendix*, Fig. S9*A*). Indeed, exclusion of the six outliers with >1% contamination by CD4^−^ T cells did alter the pattern of our observations for the CD4^+^ fraction (*SI Appendix*, Fig. S9*B*). Predictably, the CD4^−^ fraction was enriched for CD8^+^ T cells but had a variable degree of contamination by CD8^−^ (CD4^+^ and double-negative) T cells. Importantly, we observed no correlation in the CD4^−^ fraction between purity and clonal metrics (*SI Appendix*, Fig. S9*C*).

### TRBV Sequence Clustering in COVID-19.

We next sought evidence for repertoire changes imposed by antigen-specific responses to SARS-CoV-2. Because the TCRs of conventional αβ T cells are major histocompatibility complex (MHC) restricted, we first used DNA typing to identify discrete MHC class I (*n* = 21) and class II alleles (*n* = 28) shared across at least eight individuals, and for each of those HLA alleles, we then assessed TRBV-CDR3-J sequences (>2 million unique CD4^+^ TRB sequences; ∼900,000 unique CD8^+^ TRB sequences) for clustering (*SI Appendix*, Table S5). As an example, 43,957 CD8^+^ TRB sequences were contributed by 9 HLA-B*07:02^+^ SARS-CoV-2–exposed individuals, while 81,342 CD8^+^ TRB sequences were contributed by 11 HLA-B*07:02^+^ sero(–) individuals (Fig. 3*D*). Given the diversity of HLA alleles of individuals in our study, it was not possible to match all alleles for each clustering run. Thus, we could not unequivocally attribute all of the aforementioned CD8^+^ TRB sequences to HLA-B*07:02–restricted T cells, although it seems logical to assume that the sequences will be highly enriched in those contributing to HLA-B*07:02–restricted TCRs. Therefore, we proceeded to cluster the sequences using GLIPH2 (38), which infers similar peptide-MHC specificities from the structural and biochemical properties predicted for discrete amino acid sequences and their close relatives (*SI Appendix*, *Materials and Methods*).

If clusters are driven in large part by antigen-specific TCRs responsive to virus infection, then their composition would be anticipated to be contributed to primarily by SARS-CoV-2–exposed individuals. One such cluster is shown for HLA-B*07:02^+^ individuals (Fig. 3*D*), with sequences contributed by 6 of 9 SARS-CoV-2–exposed individuals and by only 1 of 11 sero(–) HLA-B*07:02^+^ individuals (*P* < 0.05 by Fisher’s exact test). Clustering of TCRs was performed for every HLA allele for which we had a sufficient number of donors in SARS-CoV-2–exposed and –unexposed cohorts. Most clusters (*n* = ∼25,000) contained similar representation from both cohorts, attesting to the scope of the TCRβ repertoire beyond any impact of SARS-CoV-2. Nonetheless, across all HLA alleles assessed, >3,000 clusters (*n* = 2,993 clusters, containing 21,869 CD4^+^ TCRs; *n* = 511 clusters containing 3,458 CD8^+^ TCRs) were significantly overcontributed to by SARS-CoV-2–exposed individuals (Fisher’s exact test; *SI Appendix*, Table S5), whereas only one single cluster (in the CD4^+^ compartment) showed significant over-representation of sero(–) individuals (*SI Appendix*, Table S5). Despite the exaggerated TRB repertoire focusing in older patients (Fig. 3 *B* and *C*), the proportions of unique TRB repertoires that were found in clusters were comparable for SARS-CoV-2–exposed individuals aged <50 y and those aged ≥50 y (*SI Appendix*, Fig. S10 *A* and *B*). Hence, the older patients achieve an equivalent end point but set against a more disruptive impact on the global repertoires, presumably reflecting greater expansions of TCRs within clusters and/or expansions of clones outside of clusters that might reflect dysregulation of T cells that are not virus specific.

### TRBV Antigen Specificity in COVID-19.

While TRB sequences do not alone determine specificity, they commonly contribute key signatures of antigen reactivity, a fact that underpins GLIPH2-based clustering. Thus, focusing on donors positive for HLA-A*02:01, the most frequently encountered HLA allele among our SARS-CoV-2–exposed and control cohorts, we asked whether their TCRs overlapped with 344 TCRs reported to bind SARS-CoV-2 spike peptide YLQPRTFLL presented by HLA-A*02:01 (39). Indeed, we identified an overlap of 12 TRB sequences that was highly specific to both virus exposure and HLA background (i.e., it occurred in 6 of 17 HLA-A*02:01^+^SARS-CoV-2–exposed individuals, but in none of 21 HLA-A*02:01^+^ sero(–) individuals, and in no SARS-CoV2–exposed individuals who were not HLA-A*02:01^+^) (Fig. 3*E*). Although this degree of overlap might seem slight, it was greater than the overlap reported when two different assays (tetramer-staining versus cytokine-release assays) were used to identify YLQPRTFLL-reactive T cells in the primary source study (39). The relatively private nature of the αβ TCR response is evidenced by only 1 of the 12 overlapping TCRs being detected in more than one donor.

### Age-Related Adaptive Vδ1 Cell Responses.

We next asked whether age-related impacts on the αβ T cell compartment were mirrored by γδ T cells that compose a second T cell lineage (40) with the potential to make adaptive responses following SARS-CoV-2 exposure (16, 24, 27). In fact, there have been very few opportunities to examine human γδ TCR–repertoire responses to defined infections encountered within a known period. Operationally, human γδ T cells are typically viewed as two distinct subtypes. Vδ1^+^ T cells use the *TRDV1* gene, are relatively rare in blood by comparison with extralymphoid tissues, and can seemingly make adaptive clonal expansions, albeit with uncertainty as to the driving antigens. Vδ2^+^ T cells use the *TRDV2* gene and are regarded as innate-like, based on their polyclonal responses to phospho-antigens (PAgs), which are low-molecular-mass metabolic intermediates of microbes and of virus-infected cells.

**Fig. 4.**
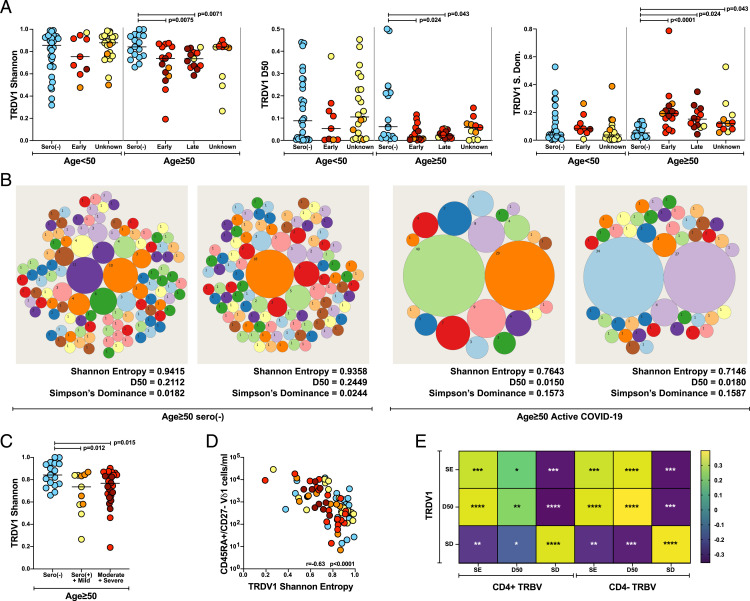
Age-related focusing of *TRDV1* repertoire in SARS-CoV-2–exposed donors. (*A*) Overall TRDV1 repertoire diversity assessed by Shannon entropy, D50, and Simpson’s dominance (S dom). Sample numbers by age: <50 y: sero(–) (*n* = 30); <50 y: early (*n* = 9); <50 y: unknown (*n* = 22); ≥50 y: sero(–) (*n* = 17); ≥50 y: early (*n* = 15); ≥50 y: late (*n* = 13); and ≥50 y: unknown (*n* = 11). The bar indicates the median value. Kruskal-Wallis test with post hoc Dunn’s test against age-matched sero(–) control individuals was used for analysis; the unadjusted *P* values are shown. (*B*) Representative tree maps of *TRDV1* repertoire from two seronegative individuals aged ≥50 y (*Left* two panels) and two individuals aged ≥50 y with active COVID-19 (*Right* two panels). Each circle represents a unique clone, and the size of the circle is proportional to the size of the clone (numbers = clone count). (*C*) *TRDV1* repertoire focusing assessed by Shannon entropy in samples from individuals aged ≥50 y exposed to SARS-CoV-2 plotted by severity of disease [sero(–), *n* = 17; sero(+)+mild, *n* = 11; moderate+severe, *n* = 30]. The bar indicates the median. Kruskal-Wallis test with post hoc Dunn’s test against age-matched sero(–) control individuals was used for analysis; the unadjusted *P* values are shown. (*D*) Correlation of *TRDV1* repertoire focusing (loss of Shannon entropy) and absolute numbers of CD45RA^+^/CD27^−^ Vδ1 cells per milliliter of blood assayed by the COVID-IP Study (*n* = 81). Only samples with >30 *TRDV1* CDR3s were analyzed for repertoire diversity and plotted. Spearman correlation was used for analysis. (*E*) Correlation of *TRDV1* repertoire focusing, Shannon entropy (SE), D50, and Simpson’s dominance (SD) with CD4^+^ and CD4^−^ TRB focusing. Only samples with >30 *TRDV1* CDR3s were analyzed for repertoire diversity and plotted. The color scale denotes Spearman’s *r*. Significant correlations are denoted with asterisks. Unadjusted *P* values are displayed. *P < 0.05, **P < 0.01, ***P < 0.001, ****P < 0.0001.

As for the αβ T cell response, we again observed significant reductions in Shannon entropy and D50 and an increase in Simpson’s dominance for *TRDV1* sequences in SARS-CoV-2–exposed individuals aged ≥50 y, but not for those aged <50 y, relative to age-matched sero(–) control individuals (Fig. 4 *A* and *B*). This focusing could be graphically illustrated by tree maps, where each unique clone is represented by a circle and the size of the circle represents the number of copies of that clone; thus, tree maps with evenly sized circles are polyclonal, while those dominated by a few large circles are clonally focused and oligoclonal (Fig. 4*B*).

Age-related *TRDV1* clonal focusing was detected in early samples and was sustained in late samples. Perhaps reflective of this durability, significant focusing was also detected (by Simpson’s dominance) among sero(+) samples (Fig. 4*A*), and it was seen in individuals with active COVID-19 regardless of disease severity (Fig. 4*C*). These observations argue that *TRDV1* focusing was most likely driven by virus exposure, consistent with which the decrease in *TRDV1* Shannon entropy was significantly and strongly correlated with the expansion of CD45RA^+^CD27^−^ Vδ1^+^ T cells (Fig. 4*D*), which emerged as one of only two immunological correlates of virus titers in the COVID-IP study, the other being natural killer cell numbers (16). Clonal expansions of human CD45RA^+^CD27^−^ Vδ1^+^ cells have been observed in the blood in several settings, including cytomegalovirus (CMV) reactivation (41–44), but this is seemingly the first clear example of such an adaptive response in relation to acute viral infection.

There was a statistically significant correlation of *TRDV1* focusing with CD4^+^ and CD8^+^ TRB focusing (Fig. 4*E*), but in contrast to TRB or IGH sequences (Fig. 3*E* and *SI Appendix*, Table S3), very few *TRDV1* sequences were shared across donors, and none was significantly enriched in SARS-CoV-2–exposed individuals, suggesting that the Vδ1 responses in COVID-19 are almost exclusively private. Likewise, very few public sequences were reported in other settings of Vδ1 focusing (42). Likely, Vδ1^+^ TCRs are mostly not specific for pathogen-derived antigens but react to myriad molecular sentinels of dysregulation directly resulting from virus infection. Indeed, the CDR3 lengths, which can be highly variable for *TRDV1*, showed comparable distributions across SARS-CoV-2–exposed individuals, sero(–) individuals, and a prior analysis of human breast-resident Vδ1 TCRs (45) (*SI Appendix*, Fig. S11).

### Age-Related Vδ2 Cell Losses.

**Fig. 5.**
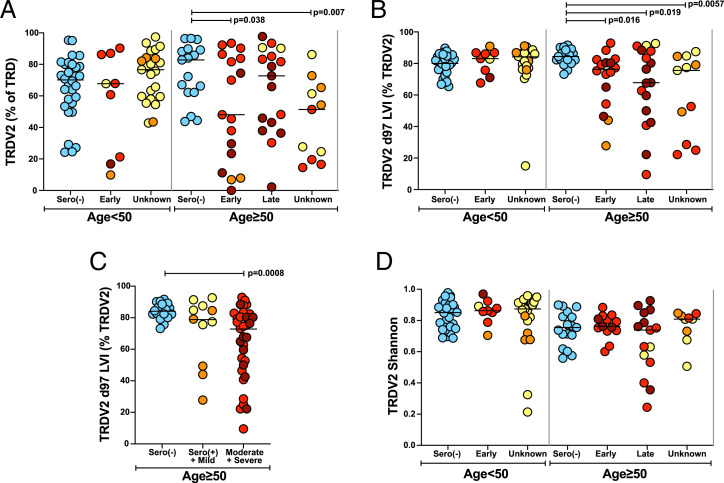
Age-related selective depletion of Vδ2 T cells in SARS-CoV-2–exposed donors. (*A*) Vδ2 T cells (TRDV2) as a percentage of total γδ T cells (TRD). (*B*) d97-LVI PAg-reactive *TRDV2* sequences recovered as a percentage of total *TRDV2* sequences recovered. (*A* and *B*) Age: <50 y: sero(–) (*n* = 30); <50 y: early (*n* = 9); <50 y: unknown (*n* = 23); ≥50 y: sero(–) (*n* = 17); ≥50 y: early (*n* = 16); ≥50 y: late (*n* = 17); and ≥50 y: unknown (*n* = 11). (*C*) d97-LVI PAg-reactive *TRDV2* sequences recovered as a percentage of total *TRDV2* sequences recovered plotted by severity of COVID-19 disease [sero(–), *n* = 17; sero(+)+mild, *n* = 11; moderate+severe, *n* = 33]. (*D*) Overall *TRDV2* repertoire diversity assessed by Shannon entropy. Only samples with >30 *TRDV2* sequences were analyzed for repertoire diversity and plotted. The bar indicates the median. Kruskal-Wallis test with post hoc Dunn’s test against age-matched sero(–) control individuals was used for analysis; the unadjusted *P* values are shown. Age: <50 y: sero(–) (*n* = 30); <50 y: early (*n* = 9); <50 y: unknown (*n* = 23); ≥50 y: sero(–) (*n* = 17); ≥50 y: early (*n* = 13); ≥50 y: late (*n* = 16); and ≥50 y: unknown (*n* = 9).

Consistent with the prominence of Vδ2^+^ T cells in peripheral blood, most TRD CDR3s were *TRDV2*-D-J rearrangements in sero(–) individuals of all ages tested. This was also true for SARS-CoV-2–exposed individuals aged <50 y, albeit there was interindividual variation as is well established for peripheral blood Vγ9Vδ2^+^ cells (46, 47) (Fig. 5*A*). By contrast, the contributions of Vδ2^+^ cells were significantly reduced in SARS-CoV-2–exposed individuals aged ≥50 y (Fig. 5*A*). Indeed, consistent with flow cytometry data from the COVID-IP study, >50% of patients with COVID-19 who were aged ≥50 y and sampled within 14 d of symptom onset had *TRDV2* sequences collectively accounting for less than half of all TRD sequences (Fig. 5*A*).

Interestingly, *TRDV2*-sequence depletion from the blood was highly selective. Vδ2 PAg reactivity requires the pairing of Vγ9 with Vδ2 and largely relies on a CDR3δ that includes a leucine, valine, or isoleucine residue at position 97 (d97 LVI) (48, 49). Whereas d97 LVI sequences accounted for ∼80% of the *TRDV2* sequences in sero(–) individuals, this was reduced to between 9% and 70% for many SARS-CoV-2–exposed individuals aged ≥50 y (Fig. 5*B*), whereas no such changes were evident in those aged <50 y (Fig. 5*B*). Thus, there was a highly selective, age-related depletion of blood PAg–reactive Vδ2^+^ T cells. However, by contrast to *TRDV1* focusing, age-related d97 LVI depletion correlated with disease severity, albeit it was also apparent for several individuals who had experienced mild or moderate disease (Fig. 5*C*). Unlike, Vδ1^+^ T cells, there was no consistent evidence of clonal focusing in the Vδ2^+^ T cell compartment in relation to SARS-CoV-2 exposure (Fig. 5*D*), in accordance with the frequent classification of Vδ2^+^ T cells as innate-like.

**Fig. 6.**
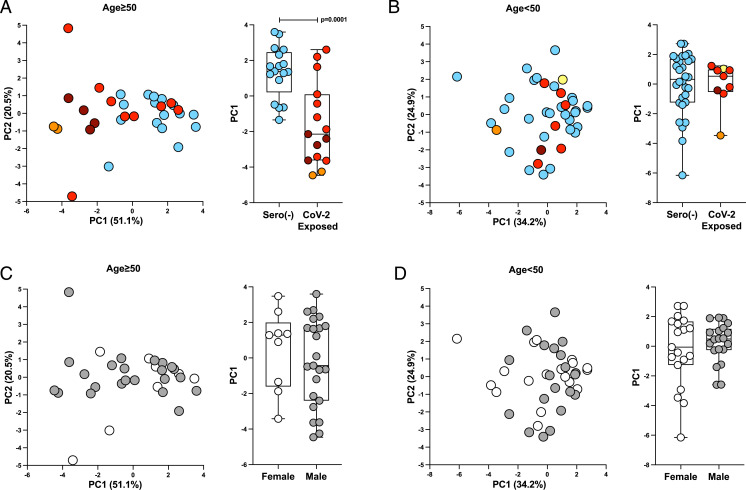
Disruptive T cell repertoire responses in SARS-CoV-2 are associated with age. Principal component analyses (PCAs) of age-related T cell repertoire responses (components included: CD4^+^ TRBV, CD4^−^ TRBV, and *TRDV1* Shannon entropy, D50, and Simpson’s dominance, and *TRDV2* % of TRD and *TRDV2* d97 LVI percentage) in sero(–) samples and SAR-CoV-2–exposed samples (restricted to the “early” cohort to avoid bias from time from symptom onset). (*A*) PCA of T cell repertoire responses in samples from donors aged ≥50 y (*Left*) colored by severity, and box plot of principle component (PC) 1 split by SARS-CoV-2 exposure (*Right*), demonstrating a significant difference in PC1 between cohorts. (*B*) PCA of T cell repertoire responses in samples from donors aged <50 y (*Left*) colored by severity, and box plot of PC1 split by SARS-CoV-2 exposure (*Right*), demonstrating no significant difference in PC1 between cohorts. (*C*) PCA of T cell repertoire responses in samples from donors aged ≥50 y (*Left*) shaded by sex, and box plot of PC1 split by sex (*Right*), demonstrating no significant difference in sex composition. (*D*) PCA of T cell repertoire responses in samples from donors aged <50 y (*Left*) shaded by sex, and box plot of PC1 split by sex (*Right*). Mann–Whitney *U* test was used for analysis.

In sum, the application of immunoPETE to concurrently sequence the global IGH, TRB, and TRD repertoires has revealed several notable traits of COVID-19, including significant, generalizable IGH-repertoire focusing, possibly as anticipated. By contrast, SARS-CoV-2–associated global changes to the αβ and γδ T cell repertoires were predominantly seen in individuals aged ≥50 y (Fig. 6 *A* and *B*) with no evidence of association with sex (Fig. 6 *C* and *D*), another major risk factor for severe COVID-19.

## Discussion

In this study, we have applied a protocol for gDNA-based antigen receptor sequencing to assess at-scale the concurrent impacts of infection on the IGH, TRB, and TRD repertoires. Being gDNA based, our approach provided information about cell numbers that strongly correlated with flow cytometry data and may, therefore, prove powerful for samples such as postmortem formalin-fixed, paraffin-embedded tissues, that are refractory to other forms of cell enumeration. Erroneous sequence calls were greatly reduced by including UMIs, which is particularly important in relation to template-independent CDR3 sequences and somatic mutation in Ig genes.

Aside from vaccination, there have been few opportunities to measure the establishment of adaptive immune responses to a known challenge incurred at a reasonably well-defined time point. Indeed, there have been few opportunities to measure the architectures of such responses in the context of overt immune dysregulation, sometimes in life-threatening settings, as was the case for some of the patients in the COVID-IP study from whom we obtained antigen-receptor sequences (16). It is, therefore, noteworthy and encouraging that despite such multifaceted immune dysregulation, extensive and dynamic sequence clustering in the IGH and TRB repertoires, together with some overlap with SARS-CoV-2–associated sequences and some level of sequence sharing, albeit minor, was strongly suggestive of virus antigen–specific responses across age groups and across disease severity. Thus, immunoPETE has demonstrated robust characterization of antigen repertoires in real-world clinical samples.

In the face of the continuing COVID-19 pandemic, vaccination and serological monitoring are backbones of the public health response, in which regard it is noteworthy that B cell repertoire dynamics were largely comparable across age groups and severity indices. By contrast, the T cell compartments (i.e., αβ and γδ) unexpectedly diverged from the B cell compartment in demonstrating markedly different repertoire responses to COVID-19 in those aged ≥50 y versus those aged <50 y. This evokes flow cytometric and functional evidence for age-related chaotic dysregulation in the adaptive immune compartment in COVID-19 (50). Unfortunately, a limitation of this study was the rarity of longitudinal samples obtained at equivalent sampling intervals or at equivalent points after symptom onset. Thus, there was insufficient power to detect any clear trajectories of high-abundance clones collectively composing the top 1%.

Given that age remains one of the most significant risk factors for infectious-disease severity, even after correction for age-related comorbid conditions (7), one may consider whether age-associated exaggerations of TRB and/or TRD repertoire focusing may be contributory factors. By definition, exaggerated focusing would limit T cell diversity that may be particularly important vis-à-vis antibody-escape variants of concern and/or other coinfections. Additionally, exaggerated focusing may include disproportionate bystander expansions with potential to be immunopathogenic. In COVID-19, these TCR-repertoire disruptions were also set against a backdrop of marked age-related losses of Vδ2 sequences required to maintain innate-like responsiveness to PAgs that are a sentinel of infection by myriad bacteria and viruses. Collectively, these points are particularly germane to a scenario by which COVID-19 is becoming endemic and, as the world emerges from a zero-COVID policy, the burden of morbidity will fall disproportionately on the elderly.

A largely unresolved immunological question of clinical importance is whether adaptive responses to virus infection are limited to antigen-specific B and αβ T cells, or whether they include potentially protective responses of human γδ T cells, for which the adaptive versus innate status has been oft debated (51–53). While Vδ1^+^ clonal expansions were reported in human blood and liver (42, 43, 54), the provoking stimuli are largely unelucidated, with data supporting and questioning, respectively, a role for CMV (42, 43). By contrast, our study has provided an unique association of bona fide adaptive Vδ1 responses with a live virus challenge, as evidenced by a correlation of decreased *TRDV1* entropy with Vδ1^+^ cell expansions that, in turn, correlated with virus load (16). However, the lack of any COVID-19–associated sequence sharing supports, prima facie, the prospect that expanded γδ cell sequences may reflect reactivity to virus-induced changes in endogenous antigen expression rather than to virus (55). This underscores the importance of assessing immunological responses beyond those that are directly SARS-CoV-2 specific.

In considering the causes of age-related T cell repertoire disruptions, it is probably inappropriate to consider that those aged ≥50 y are intrinsically T cell immunodeficient since they ordinarily harbor rich CD4^+^ and CD8^+^ T cell repertoires (10). However, those aged ≥50 y may harbor greater percentages of senescence-associated CD57^+^ CD28^−^p16^+^ T cells that are refractory to clonal expansion (56), perforce leaving responses to be dominated by larger expansions of smaller numbers of clones. Moreover, an identical outcome would result from the need to expand antigen-specific, naïve T cells in order to combat newly emerging pathogens, since the naïve T cell compartment in older persons has a very uneven geometry featuring many distinct, highly unequal expansions of private specificities (10).

An alternative, but not mutually exclusive, viewpoint is that marked clonal focusing at early time points in individuals aged ≥50 y may reflect a prompt recruitment and expansion of preexisting T cells primed to related antigens (e.g., seasonal coronaviruses) (57, 58), which those aged ≥50 y are more likely to harbor. However, whether those T cells are immunoprotective against SARS-CoV-2 remains unclear (57, 58). This evokes similar findings demonstrating that early B cell responses after SARS-CoV-2 infection were enriched in cross-reactive memory B cells, including against seasonal coronaviruses, that were of uncertain protective benefit, whereas late B cells responses were enriched in neutralizing SARS-CoV-2 RBD-specific B cells (59).

The disruptive response architectures of those aged ≥50 y would not necessitate a poor outcome; they would simply constitute a potentially significant added risk factor contributing to the upward inflection point for the probability of COVID-19–associated death for those aged ≥50 y. Note that our attempts to parse responses into other age groups failed to identify a clearer segregation of TCR responses than those between people older or younger than 50 y, respectively. Such disruptive adaptive immunity might likewise have contributed to the added risk incurred by older persons encountering SARS-CoV-1 and West Nile virus, when those infections first emerged in humans (8, 9). And it may likewise contribute to relatively poor first-dose immunogenicity in older persons and patients with cancer of vaccines against neo-antigens, including SARS-CoV-2, whereas memory T cell responses to CMV, Epstein-Barr virus, influenza, and tetanus appeared largely normal (60–62).

In sum, there are many potential hazards of reduced TCR diversity that are a collateral cost of mobilizing SARS-CoV-2/COVID-19–reactive T cell immunity in many persons aged ≥50 y. As such, this is an age-related risk factor for which clinical decision-making might be enhanced by readily available information on the status of the TRB and TRD repertoires in COVID-19 and in other infectious diseases.

## Materials and Methods

### Study Design and Participants.

Peripheral blood samples were obtained from 95 donors, including patients treated for COVID-19 at Guy’s and St. Thomas’ NHS Trust (London, UK); and both sero(+) and sero(–) healthy control individuals based at King’s College London (full cohort details are listed in *SI Appendix*, Table S1). All donors were recruited as part of the COVID-IP study (16) and had given written informed consent. This study was approved by the ethics approval board of the King's College London Infectious Diseases Biobank (REC 19/SC/0232). Paired serology, flow cytometry, cytokine assay, and SARS-CoV-2 PCR data were available for most donors through their enrollment in the COVID-IP study. Full details of study design and donor cohorts are provided in the *SI Appendix*, *Materials and Methods*.

### Magnetic Bead Separation and Generation of Antigen-Receptor Libraries Using ImmunoPETE Protocol.

Peripheral blood mononuclear cells isolated by Ficoll gradient separation were split into CD4^+^ and CD4^−^ fractions using magnetic bead separation (Miltenyi, CD4 Microbeads). Genomic DNA was isolated from both fractions and used to generate quantitative TCRβ, TCRδ, and immunoglobulin-heavy chain libraries from a single-well assay using the immunoPETE protocol from Roche Sequencing Solutions. The immunoPETE protocol combines V gene priming and extension with UMI-tagged oligos, followed by J-primer extension and library amplification of bead-purified PCR products from the initial V primed extension. The full experimental protocol is provided in the *SI Appendix*, *Materials and Methods*.

### Data Processing and Statistical Analysis.

A Roche in-house bioinformatics pipeline was used to process sequencing data with additional use of R, version 4.0.3, CRAN available packages. Prism 9 (GraphPad Software) was used for data visualization. Statistical tests were performed using either Prism 9 or R, version 4.0.3, and are specified in accompanying figure legends. All tests were two sided unless otherwise specified. Full details of data processing and analysis are provided in the *SI Appendix*, *Materials and Methods*.

## Supplementary Material

Supplementary File

## Data Availability

Sequencing data is available in the AIRR Data Commons (63) and is accessible using the iReceptor Gateway (http://gateway.ireceptor.org/) (64) with study accession ID IR-Roche-000001. Software for immunoPETE is available at https://github.com/bioinform/Daedalus (65).

## References

[1] A. Sette, S. Crotty, Adaptive immunity to SARS-CoV-2 and COVID-19. Cell 184, 861–880 (2021). 33497610 10.1016/j.cell.2021.01.007 PMC7803150

[2] E. Lavezzo ; Imperial College COVID-19 Response Team; Imperial College COVID-19 Response Team, Suppression of a SARS-CoV-2 outbreak in the Italian municipality of Vo’. Nature 584, 425–429 (2020). 32604404 10.1038/s41586-020-2488-1 PMC7618354

[3] S. Tabata , Clinical characteristics of COVID-19 in 104 people with SARS-CoV-2 infection on the Diamond Princess cruise ship: A retrospective analysis. Lancet Infect. Dis. 20, 1043–1050 (2020). 32539988 10.1016/S1473-3099(20)30482-5 PMC7292609

[4] Y. Zuo , Prothrombotic autoantibodies in serum from patients hospitalized with COVID-19. Sci. Transl. Med. 12, 3876 (2020). 10.1126/scitranslmed.abd3876 PMC7724273 33139519

[5] E. Y. Wang , Diverse functional autoantibodies in patients with COVID-19. Nature 595, 283–288 (2021). 34010947 10.1038/s41586-021-03631-y PMC13130511

[6] H. L. Dugan , Profiling B cell immunodominance after SARS-CoV-2 infection reveals antibody evolution to non-neutralizing viral targets. Immunity 54, 1290–1303.e7 (2021). 34022127 10.1016/j.immuni.2021.05.001 PMC8101792

[7] E. J. Williamson , Factors associated with COVID-19-related death using OpenSAFELY. Nature 584, 430–436 (2020). 32640463 10.1038/s41586-020-2521-4 PMC7611074

[8] J. S. M. Peiris ; HKU/UCH SARS Study Group, Clinical progression and viral load in a community outbreak of coronavirus-associated SARS pneumonia: A prospective study. Lancet 361, 1767–1772 (2003). 12781535 10.1016/S0140-6736(03)13412-5 PMC7112410

[9] C. M. Jean, S. Honarmand, J. K. Louie, C. A. Glaser, Risk factors for West Nile virus neuroinvasive disease, California, 2005. Emerg. Infect. Dis. 13, 1918–1920 (2007). 18258047 10.3201/eid1312.061265 PMC2876738

[10] Q. Qi , Diversity and clonal selection in the human T-cell repertoire. Proc. Natl. Acad. Sci. U.S.A. 111, 13139–13144 (2014). 25157137 10.1073/pnas.1409155111 PMC4246948

[11] J. J. Park, K. A. v. Lee, S. Z. Lam, S. Chen, T cell receptor repertoire signatures associated with COVID-19 severity. bioRxiv [Preprint] (2021). https://www.biorxiv.org/content/10.1101/2021.11.30.470640v1. Accessed 28 January 2022.

[12] D. Simnica , Landscape of T-cell repertoires with public COVID-19-associated T-cell receptors in pre-pandemic risk cohorts. Clin. Transl. Immunology 10, e1340 (2021). 34484739 10.1002/cti2.1340 PMC8401425

[13] A. A. Minervina , Longitudinal high-throughput TCR repertoire profiling reveals the dynamics of T-cell memory formation after mild COVID-19 infection. eLife 10, 1–17 (2021). 10.7554/eLife.63502 PMC7806265 33399535

[14] J. D. Galson , Deep sequencing of B cell receptor repertoires from COVID-19 patients reveals strong convergent immune signatures. Front. Immunol. 11, 605170 (2020). 33384691 10.3389/fimmu.2020.605170 PMC7769841

[15] C. Schultheiß , Next-generation sequencing of T and B cell receptor repertoires from COVID-19 patients showed signatures associated with severity of disease. Immunity 53, 442–455.e4 (2020). 32668194 10.1016/j.immuni.2020.06.024 PMC7324317

[16] A. G. Laing , A dynamic COVID-19 immune signature includes associations with poor prognosis. Nat. Med. 26, 1623–1635 (2020). 32807934 10.1038/s41591-020-1038-6

[17] A. B. Docherty ; ISARIC4C investigators, Features of 20 133 UK patients in hospital with covid-19 using the ISARIC WHO Clinical Characterisation Protocol: Prospective observational cohort study. BMJ 369, m1985 (2020). 32444460 10.1136/bmj.m1985 PMC7243036

[18] R. Verity , Estimates of the severity of coronavirus disease 2019: A model-based analysis. Lancet Infect. Dis. 20, 669–677 (2020). 32240634 10.1016/S1473-3099(20)30243-7 PMC7158570

[19] L. Piroth , Comparison of the characteristics, morbidity, and mortality of COVID-19 and seasonal influenza: A nationwide, population-based retrospective cohort study. Lancet Respir. Med. 9, 251–259 (2021). 33341155 10.1016/S2213-2600(20)30527-0 PMC7832247

[20] A. M. Sherwood , Deep sequencing of the human TCR and TCR repertoires suggests that TCR rearranges after and T cell commitment. Sci. Trans. Med. 3, 90ra61 (2011). 10.1126/scitranslmed.3002536 PMC4179204 21734177

[21] S. Liu , Direct measurement of B-cell receptor repertoire’s composition and variation in systemic lupus erythematosus. Genes & Immunity 18, 22–27 (2017). 28053320 10.1038/gene.2016.45

[22] W. Sui , Composition and variation analysis of the TCR β-chain CDR3 repertoire in systemic lupus erythematosus using high-throughput sequencing. Mol. Immunol. 67, 455–464 (2015). 26227771 10.1016/j.molimm.2015.07.012

[23] K. Kitaura, T. Shini, T. Matsutani, R. Suzuki, A new high-throughput sequencing method for determining diversity and similarity of T cell receptor (TCR) α and β repertoires and identifying potential new invariant TCR α chains. BMC Immunol. 17, 38 (2016). 27729009 10.1186/s12865-016-0177-5 PMC5059964

[24] F. Poccia , Anti-severe acute respiratory syndrome coronavirus immune responses: The role played by V γ 9V δ 2 T cells. J. Infect. Dis. 193, 1244–1249 (2006). 16586361 10.1086/502975 PMC7110256

[25] G. Carissimo , Whole blood immunophenotyping uncovers immature neutrophil-to-VD2 T-cell ratio as an early marker for severe COVID-19. Nat. Commun. 11, 5243 (2020). 33067472 10.1038/s41467-020-19080-6 PMC7568554

[26] B. Diao , Reduction and functional exhaustion of T cells in patients with coronavirus disease 2019 (COVID-19). Front. Immunol. 11, 827 (2020). 32425950 10.3389/fimmu.2020.00827 PMC7205903

[27] G. Rijkers, T. Vervenne, P. van der Pol, More bricks in the wall against SARS-CoV-2 infection: Involvement of γ9δ2 T cells. Cell. Mol. Immunol. 17, 771–772 (2020). 32467616 10.1038/s41423-020-0473-0 PMC7331628

[28] M. I. J. Raybould, A. Kovaltsuk, C. Marks, C. M. Deane, CoV-AbDab: The Coronavirus Antibody Database. Bioinformatics (2020) 10.1093/bioinformatics/btaa739 (Accessed 9 July 2021). PMC7558925 32805021

[29] B. J. DeKosky , In-depth determination and analysis of the human paired heavy- and light-chain antibody repertoire. Nat. Med. 21, 86–91 (2014). 25501908 10.1038/nm.3743

[30] Y. Cao , Potent neutralizing antibodies against SARS-CoV-2 identified by high-throughput single-cell sequencing of convalescent patients’ B cells. Cell 182, 73–84.e16 (2020). 32425270 10.1016/j.cell.2020.05.025 PMC7231725

[31] C. Graham , Neutralization potency of monoclonal antibodies recognizing dominant and subdominant epitopes on SARS-CoV-2 Spike is impacted by the B.1.1.7 variant. Immunity 54, 1276–1289.e6 (2021). 33836142 10.1016/j.immuni.2021.03.023 PMC8015430

[32] T. M. Ko , Immunoglobulin profiling identifies unique signatures in patients with Kawasaki disease during intravenous immunoglobulin treatment. Hum. Mol. Genet. 27, 2671–2677 (2018). 29771320 10.1093/hmg/ddy176 PMC6048982

[33] M. H. Cheng , Superantigenic character of an insert unique to SARS-CoV-2 spike supported by skewed TCR repertoire in patients with hyperinflammation. Proc. Natl. Acad. Sci. U.S.A. 117, 25254–25262 (2020). 32989130 10.1073/pnas.2010722117 PMC7568239

[34] R. A. Porritt , HLA class I-associated expansion of TRBV11-2 T cells in multisystem inflammatory syndrome in children. J. Clin. Invest. 131, e146614 (2021). 10.1172/JCI146614 PMC8121516 33705359

[35] H. Flament , Outcome of SARS-CoV-2 infection is linked to MAIT cell activation and cytotoxicity. Nat. Immunol. 22, 322–335 (2021). 33531712 10.1038/s41590-021-00870-z

[36] N. Zhang, M. J. Bevan, CD8(+) T cells: Foot soldiers of the immune system. Immunity 35, 161–168 (2011). 21867926 10.1016/j.immuni.2011.07.010 PMC3303224

[37] H. M. Li , TCRβ repertoire of CD4+ and CD8+ T cells is distinct in richness, distribution, and CDR3 amino acid composition. J. Leukoc. Biol. 99, 505–513 (2016). 26394815 10.1189/jlb.6A0215-071RR PMC5338248

[38] H. Huang, C. Wang, F. Rubelt, T. J. Scriba, M. M. Davis, Analyzing the Mycobacterium tuberculosis immune response by T-cell receptor clustering with GLIPH2 and genome-wide antigen screening. Nat. Biotechnol. 38, 1194–1202 (2020). 32341563 10.1038/s41587-020-0505-4 PMC7541396

[39] A. S. Shomuradova , SARS-CoV-2 epitopes are recognized by a public and diverse repertoire of human T cell receptors. Immunity 53, 1245–1257.e5 (2020). 33326767 10.1016/j.immuni.2020.11.004 PMC7664363

[40] A. C. Hayday , Structure, organization, and somatic rearrangement of T cell gamma genes. Cell 40, 259–269 (1985). 3917858 10.1016/0092-8674(85)90140-0

[41] J. Déchanet , Implication of γδ T cells in the human immune response to cytomegalovirus. J. Clin. Invest. 103, 1437–1449 (1999). 10330426 10.1172/JCI5409 PMC408467

[42] M. S. Davey , Clonal selection in the human Vδ1 T cell repertoire indicates γδ TCR-dependent adaptive immune surveillance. Nat. Commun. 8, 14760 (2017). 28248310 10.1038/ncomms14760 PMC5337994

[43] S. Ravens , Human γδ T cells are quickly reconstituted after stem-cell transplantation and show adaptive clonal expansion in response to viral infection. Nat. Immunol. 18, 393–401 (2017). 28218745 10.1038/ni.3686

[44] T. Rutishauser , Activation of TCR Vδ1^+^ and Vδ1^-^Vδ2^-^ γδ T cells upon controlled infection with *Plasmodium falciparum* in Tanzanian volunteers. J. Immunol. 204, 180–191 (2020). 31801816 10.4049/jimmunol.1900669

[45] Y. Wu , An innate-like Vδ1^+^ γδ T cell compartment in the human breast is associated with remission in triple-negative breast cancer. Sci. Transl. Med. 11, eaax9364 (2019). 31597756 10.1126/scitranslmed.aax9364 PMC6877350

[46] S. Esin , Different percentages of peripheral blood γ δ + T cells in healthy individuals from different areas of the world. Scand. J. Immunol. 43, 593–596 (1996). 8633219 10.1046/j.1365-3083.1996.d01-79.x

[47] S. Fonseca , Human peripheral blood gamma delta T cells: Report on a series of healthy Caucasian Portuguese adults and comprehensive review of the literature. Cells 9, 729 (2020). 10.3390/cells9030729 PMC7140678 32188103

[48] F. Davodeau , Peripheral selection of antigen receptor junctional features in a major human γ δ subset. Eur. J. Immunol. 23, 804–808 (1993). 8384559 10.1002/eji.1830230405

[49] S. Yamashita, Y. Tanaka, M. Harazaki, B. Mikami, N. Minato, Recognition mechanism of non-peptide antigens by human gammadelta T cells. Int. Immunol. 15, 1301–1307 (2003). 14565928 10.1093/intimm/dxg129

[50] C. Rydyznski Moderbacher , Antigen-specific adaptive immunity to SARS-CoV-2 in acute COVID-19 and associations with age and disease severity. Cell 183, 996–1012.e19 (2020). 33010815 10.1016/j.cell.2020.09.038 PMC7494270

[51] M. S. Davey, C. R. Willcox, A. T. Baker, S. Hunter, B. E. Willcox, Recasting human Vδ1 lymphocytes in an adaptive role. Trends Immunol. 39, 446–459 (2018). 29680462 10.1016/j.it.2018.03.003 PMC5980997

[52] B. E. Willcox, C. R. Willcox, γδ TCR ligands: The quest to solve a 500-million-year-old mystery. Nat. Immunol. 20, 121–128 (2019). 30664765 10.1038/s41590-018-0304-y

[53] A. C. Hayday, P. Vantourout, The innate biologies of adaptive antigen receptors. Annu. Rev. Immunol. 38, 487–510 (2020). 32017636 10.1146/annurev-immunol-102819-023144

[54] S. Hunter , Human liver infiltrating γδ T cells are composed of clonally expanded circulating and tissue-resident populations. J. Hepatol. 69, 654–665 (2018). 29758330 10.1016/j.jhep.2018.05.007 PMC6089840

[55] A. C. Hayday, γδ T cell update: Adaptate orchestrators of immune surveillance. J. Immunol. 203, 311–320 (2019). 31285310 10.4049/jimmunol.1800934

[56] A. N. Akbar, S. M. Henson, A. Lanna, Senescence of T lymphocytes: Implications for enhancing human immunity. Trends Immunol. 37, 866–876 (2016). 27720177 10.1016/j.it.2016.09.002

[57] A. Grifoni , Targets of T cell responses to SARS-CoV-2 coronavirus in humans with COVID-19 disease and unexposed individuals. Cell 181, 1489–1501.e15 (2020). 32473127 10.1016/j.cell.2020.05.015 PMC7237901

[58] P. Bacher , Low-avidity CD4^+^ T cell responses to SARS-CoV-2 in unexposed individuals and humans with severe COVID-19. Immunity 53, 1258–1271.e5 (2020). 33296686 10.1016/j.immuni.2020.11.016 PMC7689350

[59] A. Sokal , Maturation and persistence of the anti-SARS-CoV-2 memory B cell response. Cell 184, 1201–1213.e14 (2021). 33571429 10.1016/j.cell.2021.01.050 PMC7994111

[60] A. Hanne-Dorthe Emborg , Vaccine effectiveness of the BNT162b2 mRNA COVID-19 vaccine against RT-PCR confirmed SARS-CoV-2 infections, hospitalisations and mortality in prioritised risk groups. medRxiv Preprint (2021). 2021.05.27.21257583) Accessed 10 Jan 2022.

[61] L. Monin-Aldama , Interim Results of the Safety and Immune-Efficacy of 1 Versus 2 Doses of COVID-19 Vaccine BNT162b2 for Cancer Patients in the Context of the UK Vaccine Priority Guidelines (Cold Spring Harbor Laboratory Press, 2021).

[62] D. A. Collier , Age-related immune response heterogeneity to SARS-CoV-2 vaccine BNT162b2. Nature 596, 417–422 (2021). 34192737 10.1038/s41586-021-03739-1 PMC8373615

[63] S. Christley, The ADC API: A Web API for the Programmatic Query of the AIRR Data Commons. Frontiers Big Data (2020). 10.3389/fdata.2020.00022. Accessed 28 July 2022. PMC7931935 33693395

[64] B. Corrie, iReceptor: A platform for querying and analyzing antibody/B-cell and T-cell receptor repertoire data across federated repositories. Immunological Reviews (2018). 10.1111/imr.12666. Accessed 28 July 2022. PMC6344122 29944754

[65] K. Luong, Daedalus, software for immunoPETE. GitHub. https://github.com/bioinform/Daedalus. Deposited 6 May 2021.

