# Supplementary material for: Global patterns of antigen receptor repertoire disruption across adaptive immune compartments in COVID-19
Source: Proc Natl Acad Sci U S A. 2022 Aug 9;119(34):e2201541119. doi: 10.1073/pnas.2201541119 (PMC9407655; doi:10.1073/pnas.2201541119)
Supplement: Supplementary File [file pnas.2201541119.sapp.pdf]

PNAS

Supplementary Information for:

Global Patterns of Antigen Receptor Repertoire Disruption Across Adaptive Immune Compartments in COVID-19

Authors: Magdalene Joseph,[1,2†] Yin Wu,[1,2,3,4,5†] Richard Dannebaum,[6†] Florian Rubelt,[6†] Iva Zlatareva,[1,2] Anna Lorenc,[1] Zhipei Gracie Du,[6] Daniel Davies,[1,7] Fernanda Kyle-Cezar,[1] Abhishek Das,[1,8] Sarah Gee,[1] Jeffrey Seow,[9] Carl Graham,[9] Dilduz Telman,[6] Clara Bermejo,[6] Hai Lin,[6] Hosseinali Asgharian,[6] Adam G. Laing,[1] Irene del Molino del Barrio,[1,5] Leticia Monin,[2] Miguel Muñoz-Ruiz,[2] Duncan R. McKenzie,[2] Thomas S. Hayday,[1] Isaac Francos-Quijorna,[10] Shraddha Kamdar,[1] Richard Davis,[1] Vasiliki Sofra,[1] Florencia Cano,[2] Efstathios Theodoridis,[1] Lauren Martinez,[11] Blair Merrick,[12] Karen Bisnauthsing,[11] Kate Brooks,[11] Jonathan Edgeworth,[9,12] John Cason,[13] Christine Mant,[13] Katie J. Doores,[9] Pierre Vantourout,[1] Khai Luong,[6] Jan Berka,[6] and Adrian C. Hayday[1,2*]

Corresponding author: Adrian C. Hayday
Email: Adrian.Hayday@kcl.ac.uk or Adrian.Hayday@crick.ac.uk

This PDF file includes:

Supplementary Figures S1 to S11 (pages 2-23)
Supplementary Tables S1 to S5 (pages 24-29)
Supplementary Materials and Methods (pages 30-37)
References (page 37)
Key Reagents and Resources Table (page 38)

Figure S1

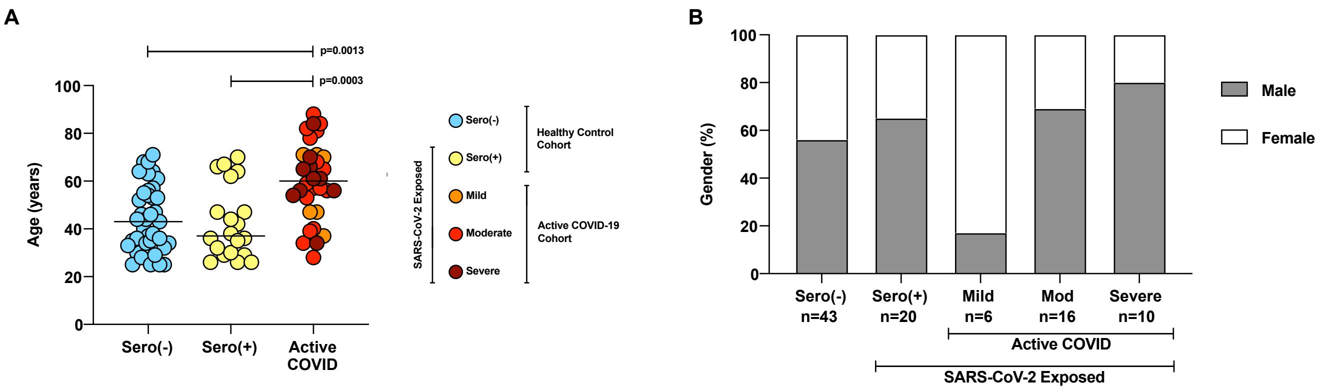
A
p=0.0013
p=0.0003
Age (years)
100
80
60
40
20
0
Sero(-)
Sero(+)
Active COVID
Sero(-)
Sero(+)
Mild
Moderate
Severe
Healthy Control Cohort
Active COVID-19 Cohort
SARS-CoV-2 Exposed
B
Gender (%)
Male
Female
Sero(-) n=43
Sero(+) n=20
Mild n=6
Mod n=16
Severe n=10
Active COVID
SARS-CoV-2 Exposed

Figure S1. Donor demographics and sequencing workflow. (A) Age in years of donors at the time of baseline blood sampling [sero(-) (n=43), sero(+) (n=20), active COVID (n=32)]. Bar = median. Kruskal-Wallis followed by post-hoc Dunn's test corrected for multiple testing. **(B)** Summary of gender proportions in study cohorts.

Figure S2

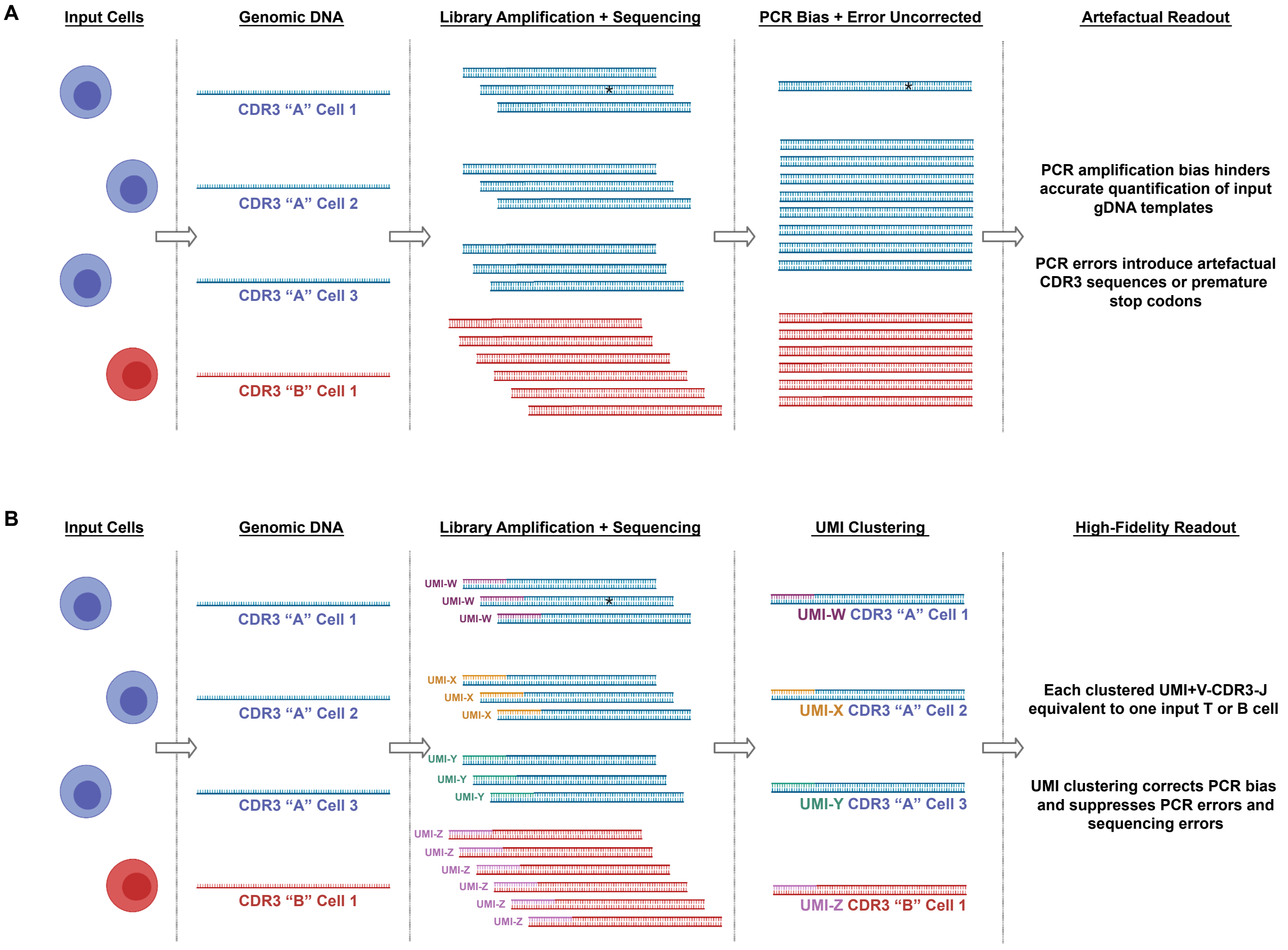

ImmunoPETE

Figure S2. Antigen receptor sequencing by immunoPETE enables high-fidelity quantitative recovery of antigen receptor sequences. (A) NGS approaches to antigen receptor sequencing are subject to PCR biases and errors which may confound quantitation and high-fidelity recovery of input sequences. **(B)** ImmunoPETE addresses these issues through the use of UMIs. Genomic DNA is selectively amplified in a multiplex PCR reaction with UMI tagged primers for all known TRBV, TRBJ, IGHV, IGHJ, TRDV and TRDJ genes. Clustered reads based on identical (or near identical) UMI+V-CDR3-J reads corrects for PCR errors (*) and amplification bias allowing for quantitative and high-fidelity mapping to input cells.

Figure S3

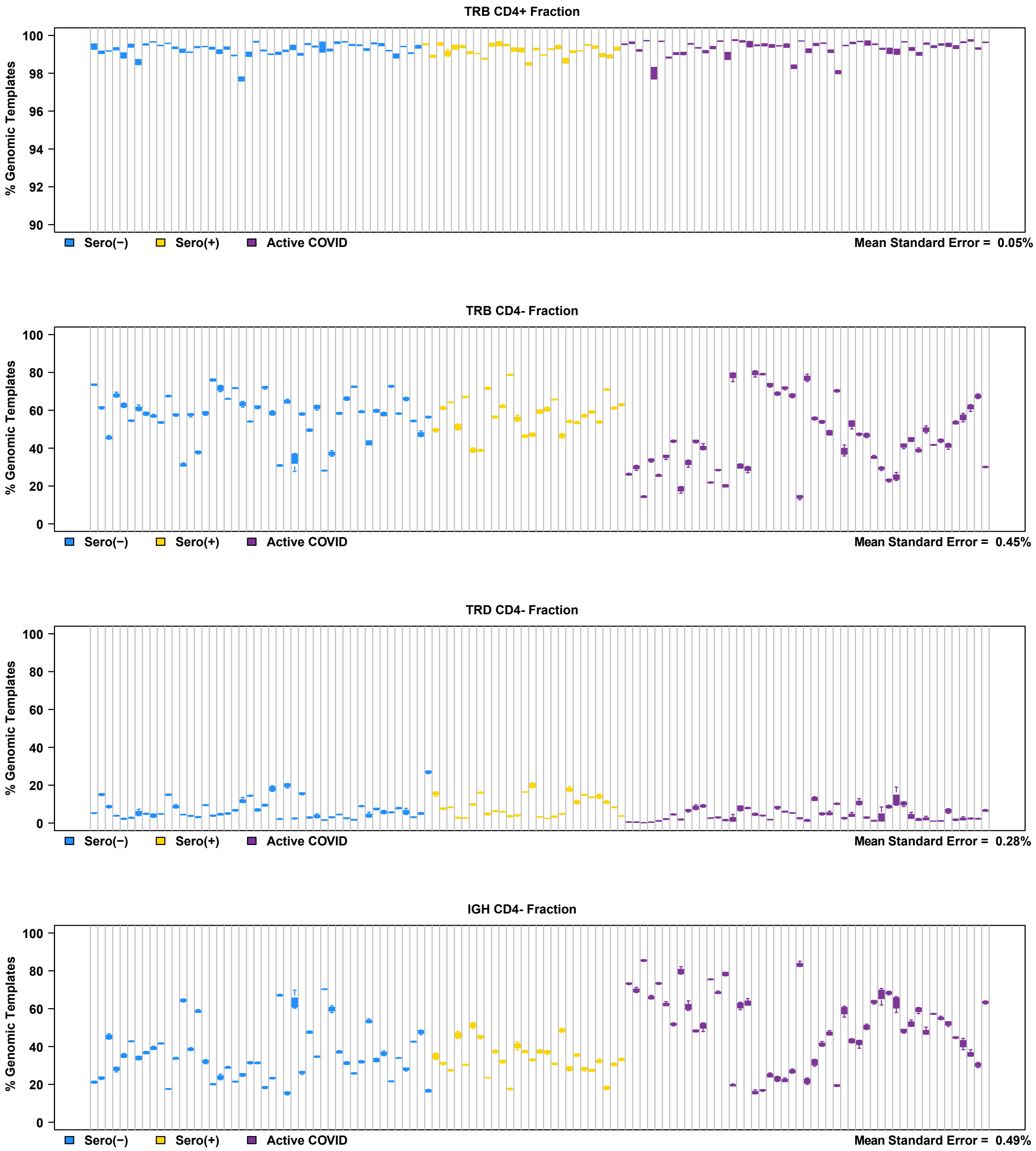

TRB CD4+ Fraction
% Genomic Templates
100
98
96
94
92
90
Sero(−)
Sero(+)
Active COVID
Mean Standard Error = 0.05%
TRB CD4- Fraction
% Genomic Templates
100
80
60
40
20
0
Sero(−)
Sero(+)
Active COVID
Mean Standard Error = 0.45%
TRD CD4- Fraction
% Genomic Templates
100
80
60
40
20
0
Sero(−)
Sero(+)
Active COVID
Mean Standard Error = 0.28%
IGH CD4- Fraction
% Genomic Templates
100
80
60
40
20
0
Sero(−)
Sero(+)
Active COVID
Mean Standard Error = 0.49%

Figure S3. ImmunoPETE demonstrates consistent recovery of input TRB, TRD and IGH templates. (A) Percentage TRB genomic templates of total productively rearranged genomic templates recovered by immunoPETE from the $CD4^{+}$ fraction. Summary data of 2 replicate libraries from each sample represented by box plots. Percentage TRB **(B)**, TRD **(C)** and IGH **(D)** genomic templates of total productively rearranged genomic templates recovered by immunoPETE from the $CD4^{-}$ fraction. Summary data of 4 replicate libraries from the $CD4^{-}$ fraction of each sample represented by box plots.

Figure S4

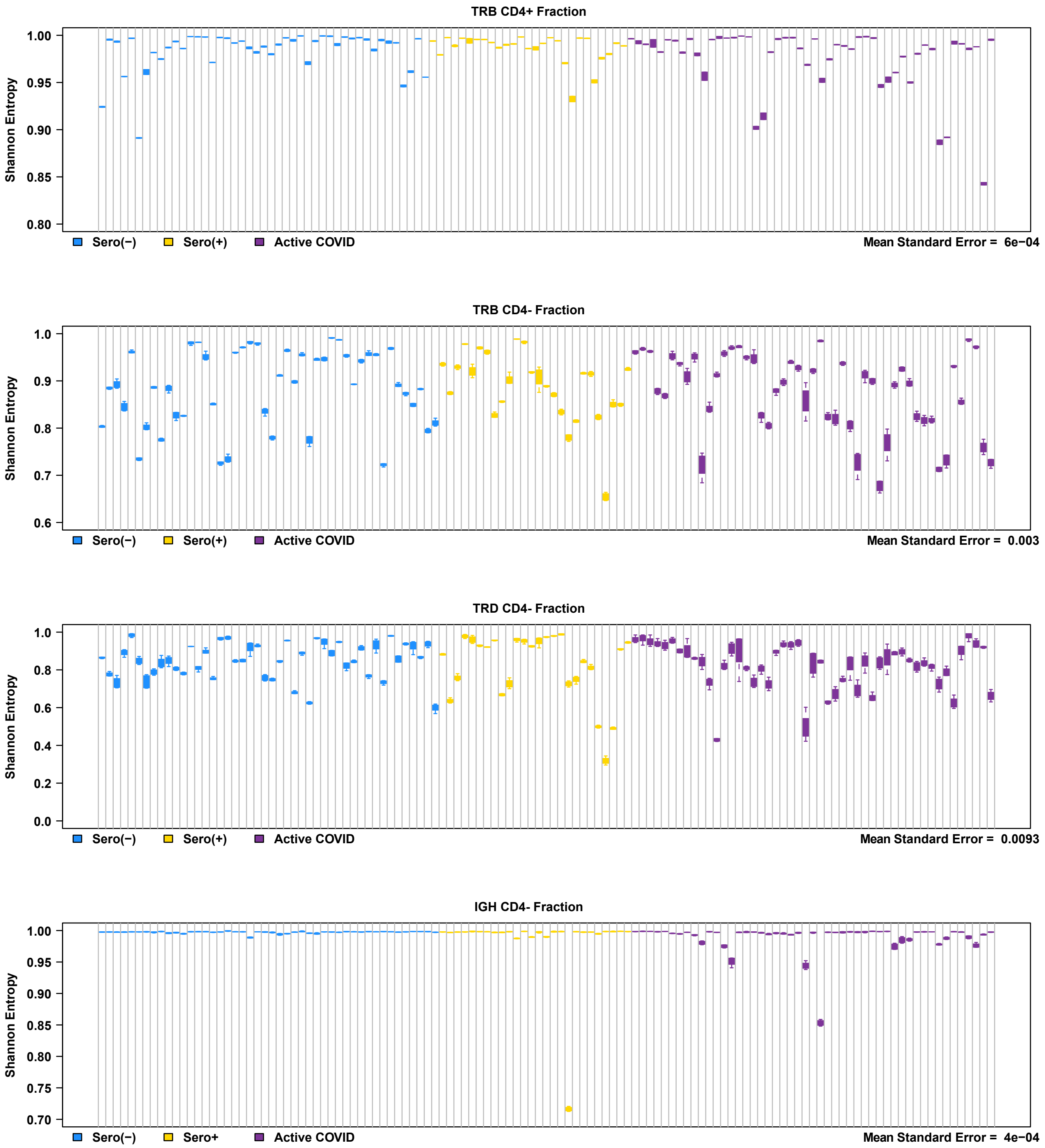
TRB CD4+ Fraction
Shannon Entropy
1.00
0.95
0.90
0.85
0.80
Sero(−)
Sero(+)
Active COVID
Mean Standard Error = 6e−04
TRB CD4- Fraction
Shannon Entropy
1.0
0.9
0.8
0.7
0.6
Sero(−)
Sero(+)
Active COVID
Mean Standard Error = 0.003
TRD CD4- Fraction
Shannon Entropy
1.0
0.8
0.6
0.4
0.2
0.0
Sero(−)
Sero(+)
Active COVID
Mean Standard Error = 0.0093
IGH CD4- Fraction
Shannon Entropy
1.00
0.95
0.90
0.85
0.80
0.75
0.70
Sero(−)
Sero+
Active COVID
Mean Standard Error = 4e−04

Figure S4. ImmunoPETE demonstrates consistent recovery of input TRB, TRD and IGH clonal architectures. **(A)** TRB Shannon entropy of $CD4^+$ fraction. Summary data of 2 replicate libraries from the $CD4^+$ fraction of each sample represented by box plots. TRB **(B)**, TRD **(C)** and IGH **(D)** Shannon entropy of $CD4^-$ fraction. Summary data of 4 replicate libraries from the $CD4^-$ fraction of each sample represented by box plots.

Figure S5

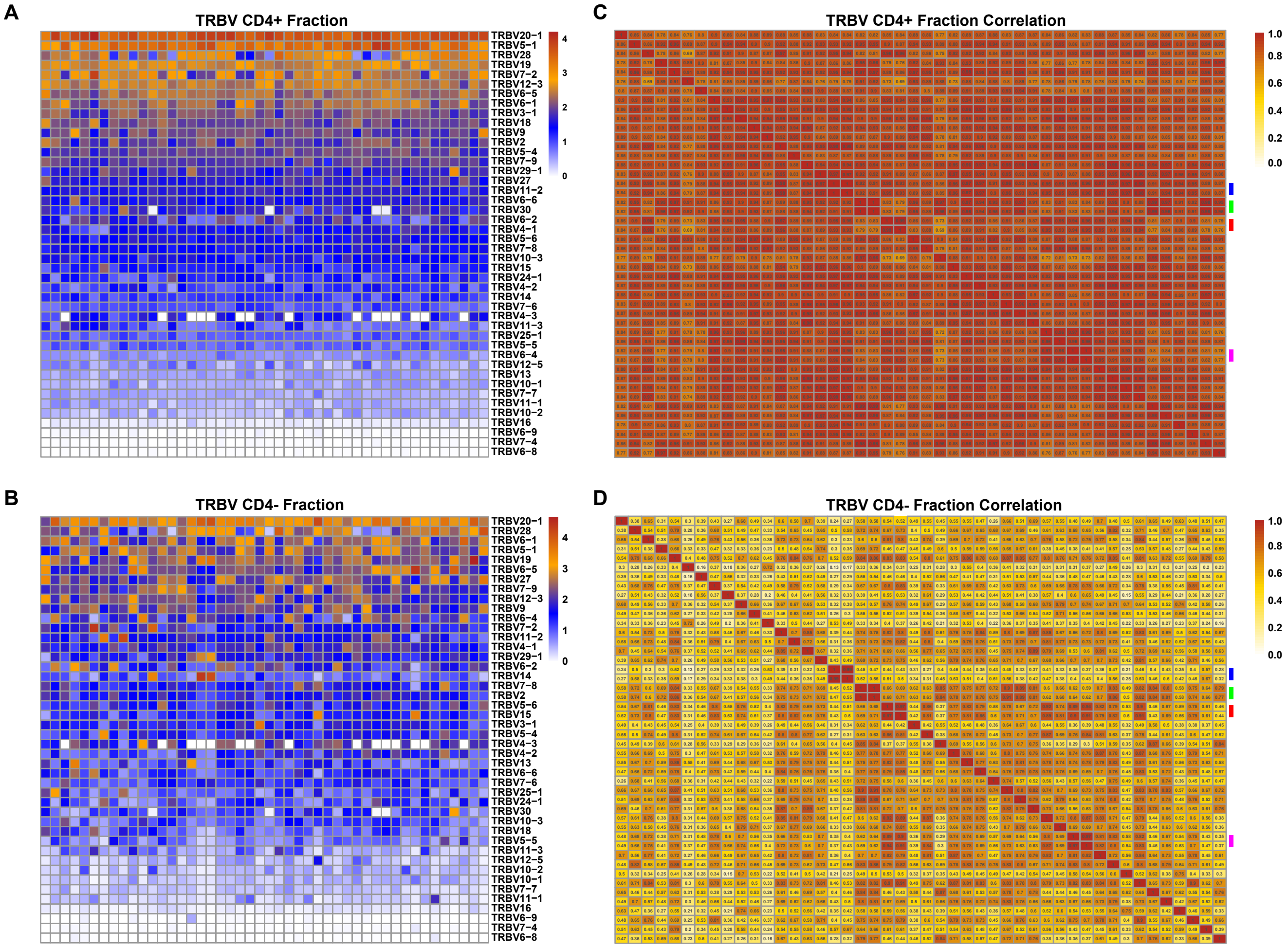
A
TRBV CD4+ Fraction
TRBV20−1
TRBV5−1
TRBV28
TRBV19
TRBV7−2
TRBV12−3
TRBV6−5
TRBV6−1
TRBV3−1
TRBV18
TRBV9
TRBV2
TRBV5−4
TRBV7−9
TRBV29−1
TRBV27
TRBV11−2
TRBV6−6
TRBV30
TRBV6−2
TRBV4−1
TRBV5−6
TRBV7−8
TRBV10−3
TRBV15
TRBV24−1
TRBV4−2
TRBV14
TRBV7−6
TRBV4−3
TRBV11−3
TRBV25−1
TRBV5−5
TRBV6−4
TRBV12−5
TRBV13
TRBV10−1
TRBV7−7
TRBV11−1
TRBV10−2
TRBV16
TRBV6−9
TRBV7−4
TRBV6−8
4
3
2
1
0
B
TRBV CD4- Fraction
TRBV20−1
TRBV28
TRBV6−1
TRBV5−1
TRBV19
TRBV6−5
TRBV27
TRBV7−9
TRBV12−3
TRBV9
TRBV6−4
TRBV7−2
TRBV11−2
TRBV4−1
TRBV29−1
TRBV6−2
TRBV14
TRBV7−8
TRBV2
TRBV5−6
TRBV15
TRBV3−1
TRBV5−4
TRBV4−3
TRBV4−2
TRBV13
TRBV6−6
TRBV7−6
TRBV25−1
TRBV24−1
TRBV30
TRBV10−3
TRBV18
TRBV5−5
TRBV11−3
TRBV12−5
TRBV10−2
TRBV10−1
TRBV7−7
TRBV11−1
TRBV16
TRBV6−9
TRBV7−4
TRBV6−8
4
3
2
1
0
C
TRBV CD4+ Fraction Correlation
1.0
0.8
0.6
0.4
0.2
0.0
D
TRBV CD4- Fraction Correlation
1.0
0.8
0.6
0.4
0.2
0.0

Figure S5. ImmunoPETE demonstrates consistent recovery of all targeted TRBV genes. (A) Heatmap of all functional TRBV genes targeted by immunoPETE primers recovered from the $CD4^+$ fraction of healthy sero(-) samples. Each column represents TRBV genes from one sample. Colour scale denotes Log_2(percentage recovery of specified TRBV gene for each sample). **(B)** Heatmap of all functional TRBV genes targeted by immunoPETE primers recovered from the $CD4^-$ fraction of healthy sero(-) samples. Each column represents TRBV genes from one sample. Colour scale denotes Log_2 (percentage recovery of specified TRBV gene for each sample). **(C)** Pairwise correlations of TRBV gene recovery from the $CD4^+$ fraction of healthy sero(-) samples presented in (A) ordered by sample ID from top to bottom and left to right. Colour scale denotes Pearson r. **(D)** Pairwise correlations of TRBV gene recovery from the $CD4^-$ fraction of healthy sero(-) samples presented in (B) ordered by sample ID from top to bottom and left to right. Colour scale denotes Pearson r.

Figure S6

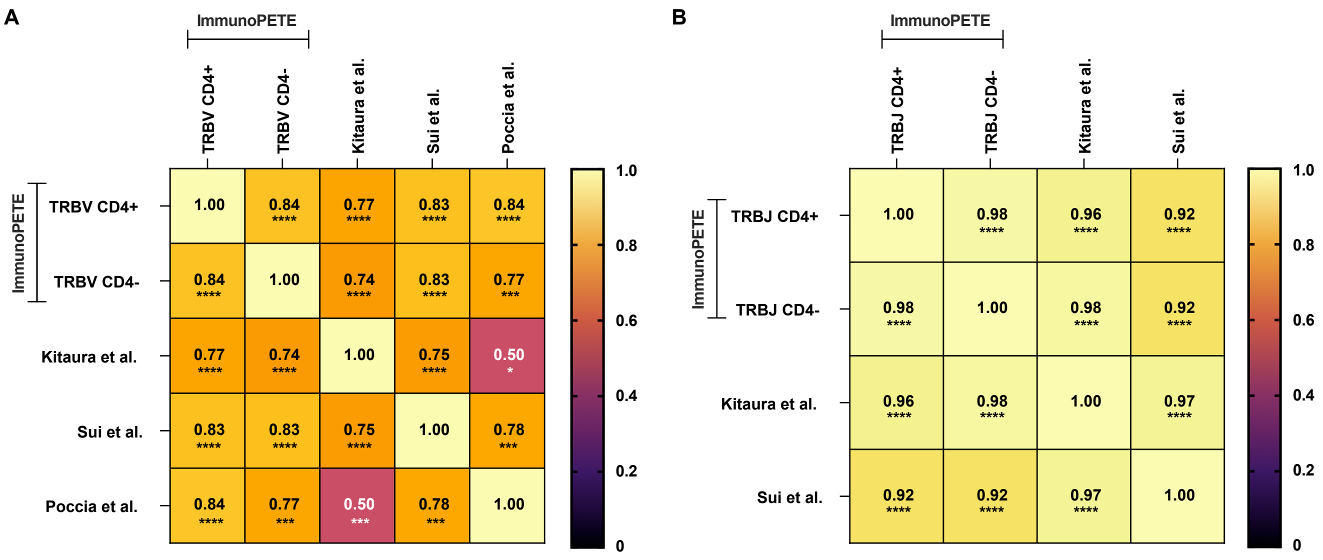
A
ImmunoPETE
TRBV CD4+
TRBV CD4-
Kitaura et al.
Sui et al.
Poccia et al.
1.00
0.84 ****
0.77 ****
0.83 ****
0.84 ****
0.84 ****
1.00
0.74 ****
0.83 ****
0.77 ***
0.77 ****
0.74 ****
1.00
0.75 ****
0.50 *
0.83 ****
0.83 ****
0.75 ****
1.00
0.78 ***
0.84 ****
0.77 ***
0.50 ***
0.78 ***
1.00
1.0
0.8
0.6
0.4
0.2
0
B
ImmunoPETE
TRBJ CD4+
TRBJ CD4-
Kitaura et al.
Sui et al.
1.00
0.98 ****
0.96 ****
0.92 ****
0.98 ****
1.00
0.98 ****
0.92 ****
0.96 ****
0.98 ****
1.00
0.97 ****
0.92 ****
0.92 ****
0.97 ****
1.00
1.0
0.8
0.6
0.4
0.2
0

Figure S6. ImmunoPETE demonstrates no gross amplification biases in TRB genes when compared with public datasets. (A) Correlation of average recovery of TRBV genes by immunoPETE from CD4+ and CD4- fractions versus published datasets (details below). **(B)** Correlation of average recovery of TRBJ genes by immunoPETE from CD4+ and CD4- fractions versus published datasets (details below). Spearman correlation r reported and denoted by colour scale. *p<0.05, **p<0.01, ***p<0.001, ****p<0.0001.

Kitaura *et al.* dataset generated using RNA sequencing with TRBC primers and without UMI correction on peripheral blood leukocyte RNA from 20 healthy adult donors. Sui *et al.* dataset generated using 45 TRBV and 13 TRBJ primers and without UMI correction on peripheral blood T cell gDNA from 13 healthy adult donors. Poccia *et al.* dataset generated using the flow cytometric "Beta Mark" assay on peripheral blood T cells from 11 healthy adult donors. Correlations to Poccia *et al.* dataset restricted to a subset of TRBV genes unambiguously identified by Beta Mark antibodies.

Figure S7

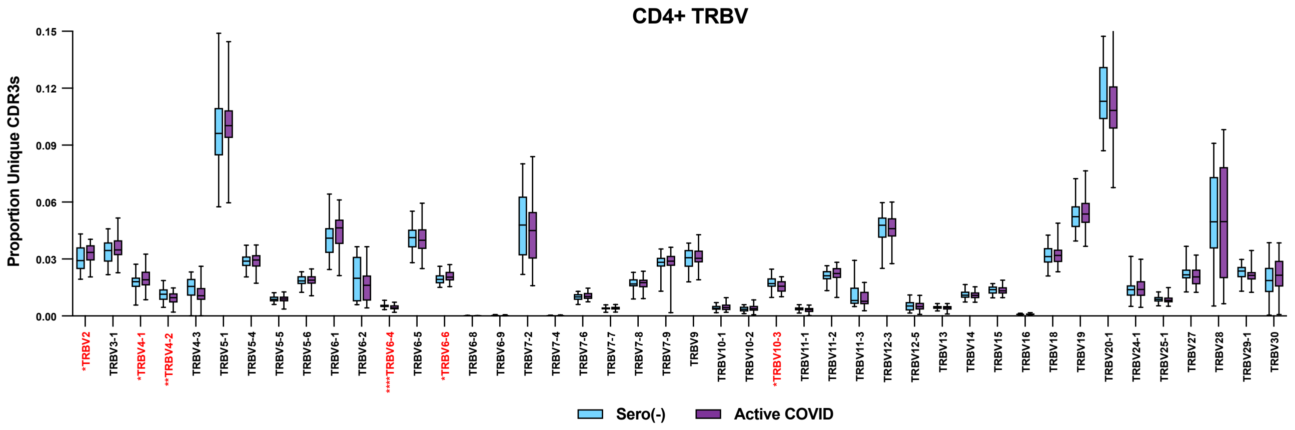
CD4+ TRBV
Proportion Unique CDR3s
0.15
0.12
0.09
0.06
0.03
0.00
*TRBV2
TRBV3-1
*TRBV4-1
**TRBV4-2
TRBV4-3
TRBV5-1
TRBV5-4
TRBV5-5
TRBV5-6
TRBV6-1
TRBV6-2
****TRBV6-4
TRBV6-5
*TRBV6-6
TRBV6-8
TRBV6-9
TRBV7-2
TRBV7-4
TRBV7-6
TRBV7-7
TRBV7-8
TRBV7-9
TRBV9
TRBV10-1
TRBV10-2
*TRBV10-3
TRBV11-1
TRBV11-2
TRBV11-3
TRBV12-3
TRBV12-5
TRBV13
TRBV14
TRBV15
TRBV16
TRBV18
TRBV19
TRBV20-1
TRBV24-1
TRBV25-1
TRBV27
TRBV28
TRBV29-1
TRBV30
Sero(-)
Active COVID

Figure S7. TRBV repertoire dynamics suggest antigen driven clonal expansions as opposed to superantigen responses. Box plots of $CD4^{+}$ TRB V gene family use as proportion of the unique CDR3s (i.e. each unique CDR3 is treated equally regardless of clone size) in samples (n=47) from sero(-) individuals and samples (n=52) from individuals with active COVID-19. TRB V genes with significantly different utilisation between cohorts highlighted in red. Median, interquartile-range and range plotted. Mann-Whitney test, unadjusted p values displayed. *p<0.05, **p<0.01, ***p<0.001, ****p<0.0001.

Figure S8

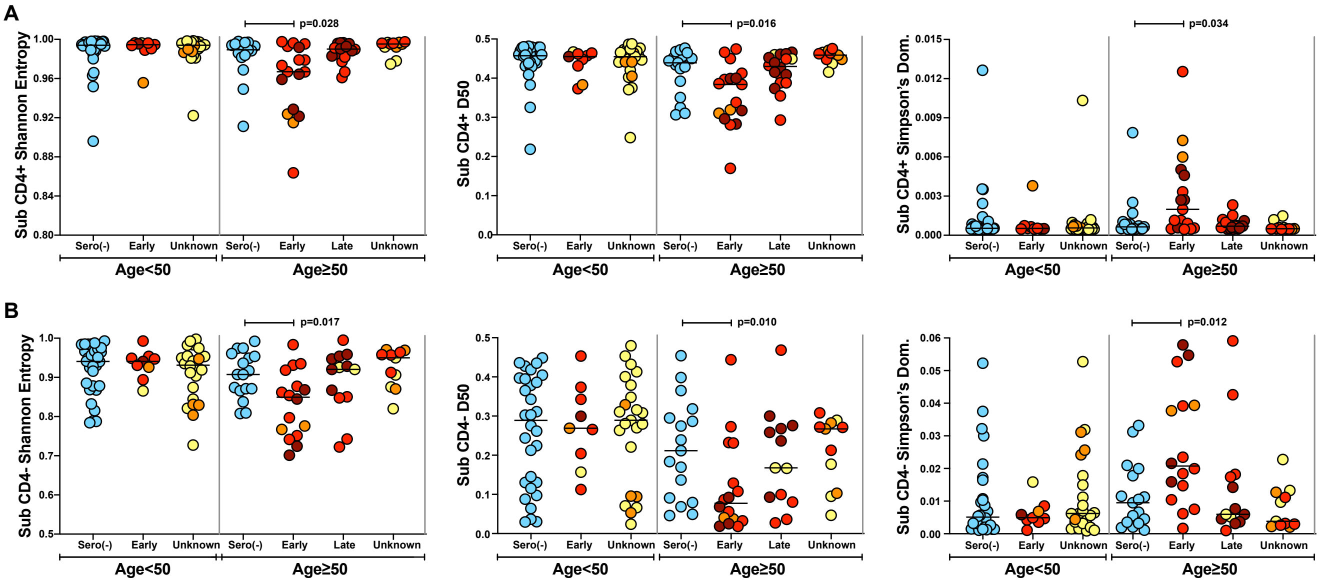
A
Sub CD4+ Shannon Entropy
p=0.028
Sub CD4+ D50
p=0.016
Sub CD4+ Simpson's Dom.
p=0.034
Sero(-)
Early
Unknown
Late
Age<50
Age≥50
B
Sub CD4- Shannon Entropy
p=0.017
Sub CD4- D50
p=0.010
Sub CD4- Simpson's Dom.
p=0.012

Figure S8. Subsampled repertoires reveal consistent age-related repertoire changes associated with COVID-19 (A) Sub-sampled (to 2400 cells) $CD4^+$ TRB repertoire diversity assessed by Shannon entropy, D50 and Simpson's dominance. For panel A: age<50 sero(-) (n=30), age<50 early (n=9), age<50 unknown (n=23), age≥50 sero(-) (n=17), age≥50 early (n=17), age≥50 late (n=18) and age≥50 unknown (n=11). **(B)** Sub-sampled (to 1200 cells) $CD4^-$ TRB repertoire diversity assessed by Shannon entropy, D50 and Simpson's dominance plotted. For panel B: age<50 sero(-) (n=30), age<50 early (n=9), age<50 unknown (n=23), age≥50 sero(-) (n=17), age≥50 early (n=16), age≥50 late (n=13) and age≥50 unknown (n=11). Bar = median. Kruskal-Wallis test with post hoc Dunn's test against age matched sero(-) control, unadjusted p values shown.

Figure S9

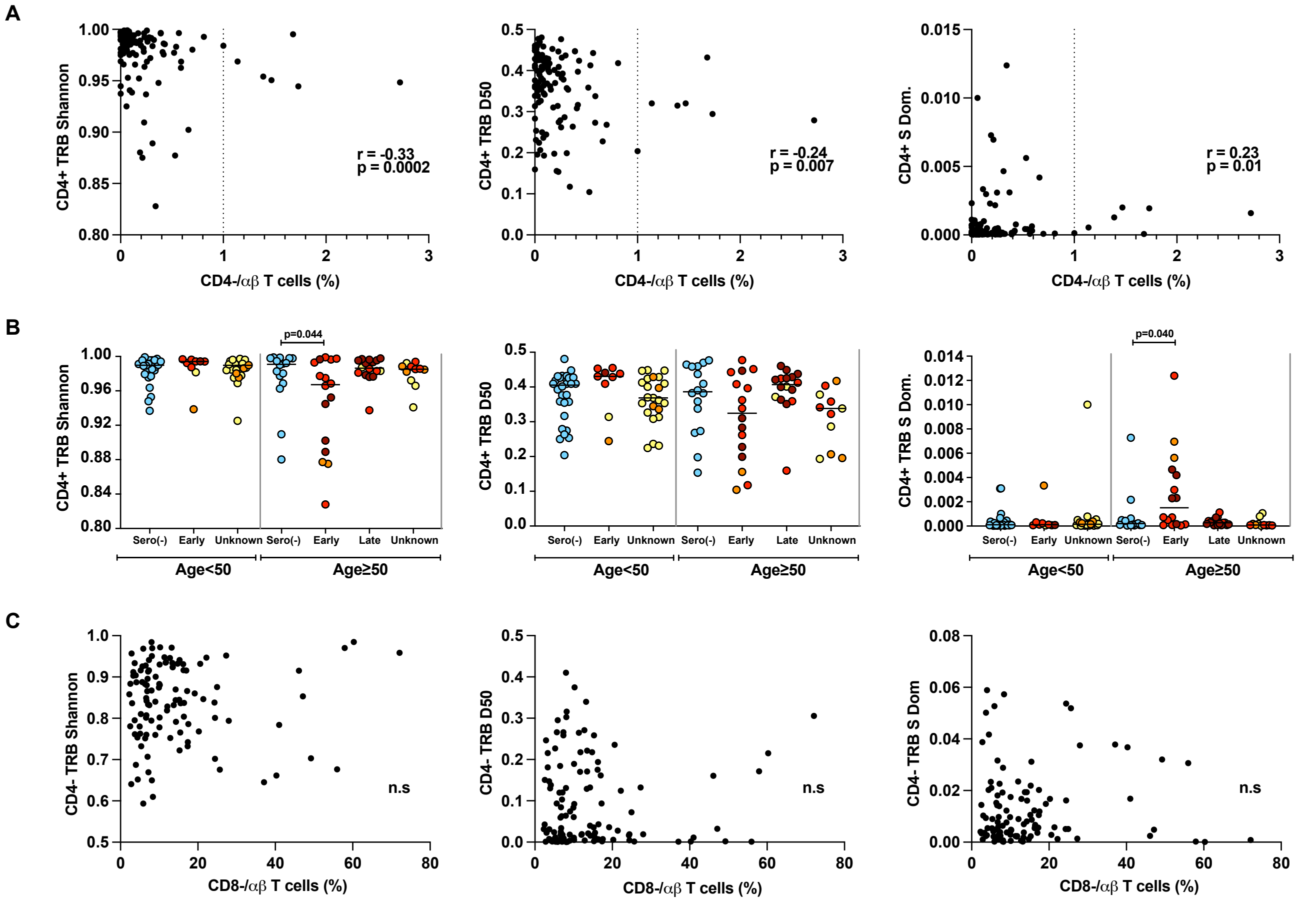

A
CD4+ TRB Shannon
CD4+ TRB D50
CD4+ S Dom.
CD4-/αβ T cells (%)
r = -0.33
p = 0.0002
r = -0.24
p = 0.007
r = 0.23
p = 0.01
B
CD4+ TRB S Dom.
p=0.044
p=0.040
Sero(-)
Early
Unknown
Late
Age<50
Age≥50
C
CD4- TRB Shannon
CD4- TRB D50
CD4- TRB S Dom
CD8-/αβ T cells (%)
n.s

Figure S9. Sorting purity of CD4$^+$ and CD4$^-$ fractions do not confound TRB metrics. (A) Correlation of CD4$^+$ TRB diversity metrics with degree of CD4$^-$ contamination of CD4$^+$ fraction (n=122). Spearman correlation. **(B)** Overall CD4$^+$ TRB repertoire diversity assessed by Shannon entropy, D50 and Simpson's dominance after excluding samples with >1% CD4$^-$ contamination of $\alpha\beta$ T cells in the CD4$^+$ fraction. Bar = median. Kruskal-Wallis test with post hoc Dunn's test against age matched sero(-) control, unadjusted p values shown. **(C)** Correlation of CD4$^-$ TRB diversity metrics with degree of CD8$^-$ contamination of CD4$^-$ fraction (n=112). Spearman correlation.

Figure S10

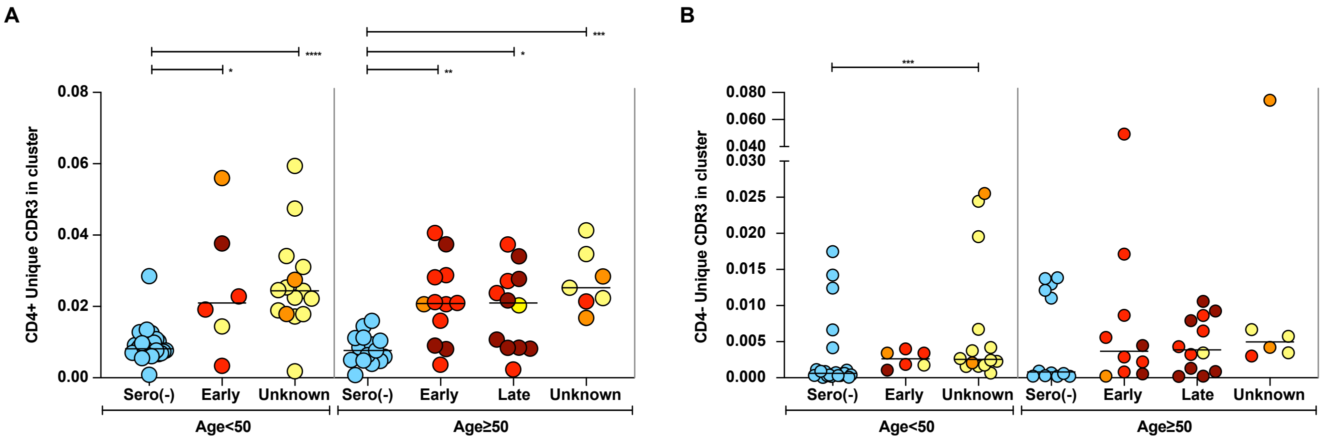
A
CD4+ Unique CDR3 in cluster
0.08
0.06
0.04
0.02
0.00
Sero(-)
Early
Unknown
Sero(-)
Early
Late
Unknown
Age<50
Age≥50
****
*
***
*
**
B
CD4- Unique CDR3 in cluster
0.080
0.060
0.040
0.030
0.025
0.020
0.015
0.010
0.005
0.000
***

Figure S10. Proportion of TRBV clustered by GLIPH2 similar between age groups. (A) Proportion of unique TCRs in clusters in CD4+ cells. Bar = median. Kruskal-Wallis test with post hoc Dunn's test against age matched sero(-) control, unadjusted p values shown. **(B)** Proportion of unique TCRs in clusters in CD4- cells. Bar = median. Kruskal-Wallis test with post hoc Dunn's test against age matched sero(-) control, unadjusted p values shown.

Figure S11

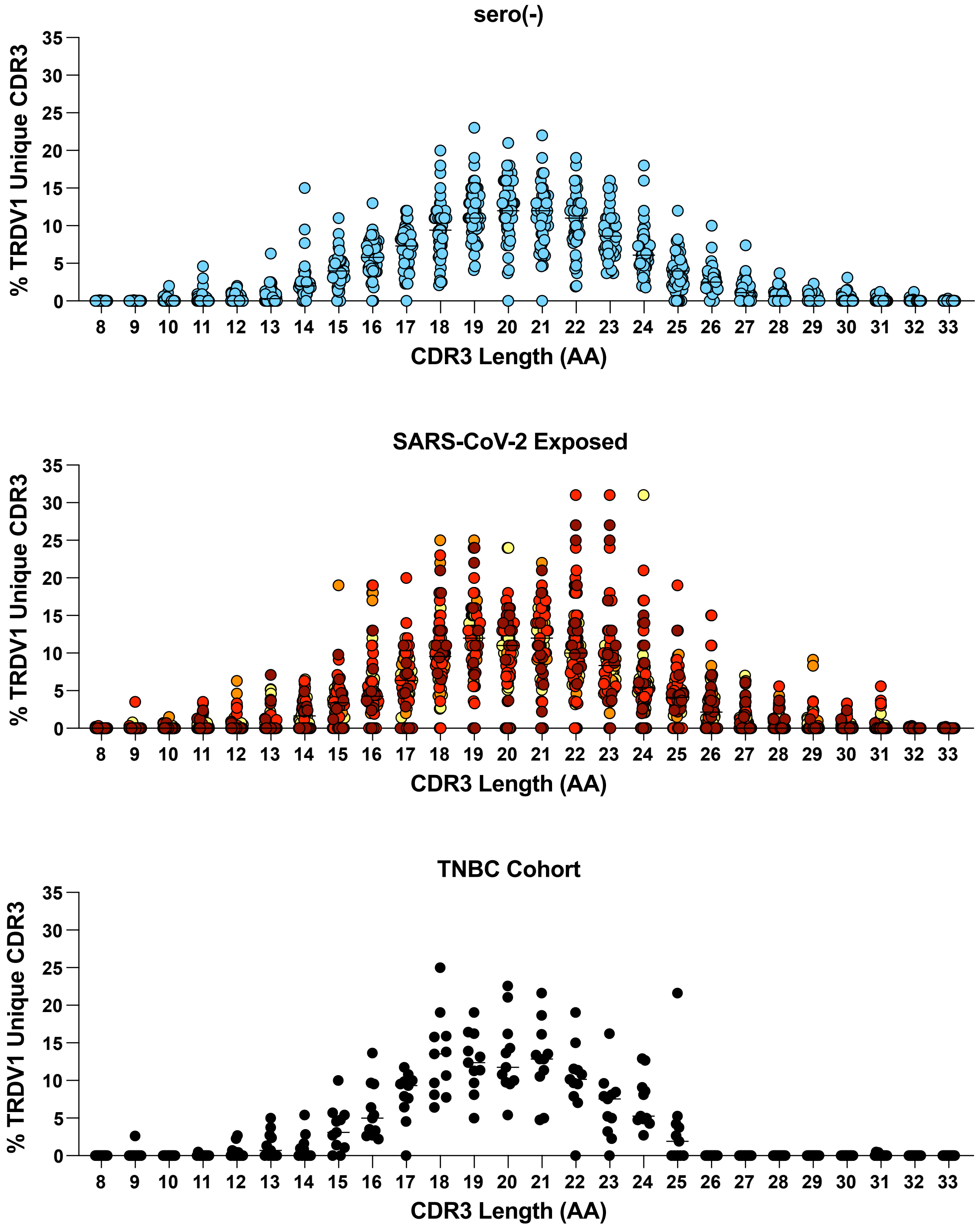

sero(-)
% TRDV1 Unique CDR3
CDR3 Length (AA)
SARS-CoV-2 Exposed
% TRDV1 Unique CDR3
CDR3 Length (AA)
TNBC Cohort
% TRDV1 Unique CDR3
CDR3 Length (AA)

Figure S11. TRDV1 CDR3 length in COVID-19. Amino acid (AA) CDR3 lengths of unique TRDV1 CDR3s plotted as a percentage of total unique TRDV1 CDR3s. Each point represents one sample. Top panel: sero(-) samples (n=47). Middle panel: SARS-CoV-2 exposed samples (sero(+) and active COVID-19 cohort) (n=70). Bottom panel: data from previous study of breast tumour infiltrating Vδ1 T cells from donors with triple-negative breast cancer (TNBC) (n=11). Only samples with >30 TRDV1 CDR3s were analysed for CDR3 length and plotted.

Patient ID	Sex	Age	Severity	SARS-CoV-2 Exposed	Active COVID-19
**HC_COV_71**	M	54	Sero(-)	NO	NO
**HC_COV_72**	F	46	Sero(-)	NO	NO
**HC_COV_73**	F	64	Sero(-)	NO	NO
**HC_COV_74**	M	34	Sero(-)	NO	NO
**HC_COV_75**	M	30	Sero(-)	NO	NO
**HC_COV_76**	F	61	Sero(-)	NO	NO
**HC_COV_77**	M	68	Sero(-)	NO	NO
**HC_COV_79**	F	57	Sero(-)	NO	NO
**HC_COV_80**	M	64	Sero(-)	NO	NO
**HC_COV_81**	F	63	Sero(-)	NO	NO
**HC_COV_82**	M	64	Sero(-)	NO	NO
**p005**	M	53	Sero(-)	NO	NO
**p019**	M	56	Sero(-)	NO	NO
**p020**	M	35	Sero(-)	NO	NO
**p021**	M	40	Sero(-)	NO	NO
**p045**	M	37	Sero(-)	NO	NO
**p050**	F	29	Sero(-)	NO	NO
**p057**	F	25	Sero(-)	NO	NO
**p060**	F	28	Sero(-)	NO	NO
**p064**	M	34	Sero(-)	NO	NO
**p065**	F	25	Sero(-)	NO	NO
**p066**	F	25	Sero(-)	NO	NO
**p069**	F	36	Sero(-)	NO	NO
**p070**	M	36	Sero(-)	NO	NO
**p077**	F	44	Sero(-)	NO	NO
**p082**	F	34	Sero(-)	NO	NO
**p085**	F	68	Sero(-)	NO	NO
**p087**	M	71	Sero(-)	NO	NO
**p089**	M	44	Sero(-)	NO	NO
**p096**	F	52	Sero(-)	NO	NO
**p100**	M	54	Sero(-)	NO	NO
**p101**	M	35	Sero(-)	NO	NO
**p107**	M	55	Sero(-)	NO	NO
**p120**	M	32	Sero(-)	NO	NO
**p123**	M	53	Sero(-)	NO	NO
**p124**	F	47	Sero(-)	NO	NO
**p125**	M	33	Sero(-)	NO	NO
**p132**	F	43	Sero(-)	NO	NO
**p138**	F	25	Sero(-)	NO	NO
**p140**	M	29	Sero(-)	NO	NO
**p151**	M	46	Sero(-)	NO	NO
**p154**	M	38	Sero(-)	NO	NO
**p155**	F	36	Sero(-)	NO	NO
**HC_COV_70**	M	47	Sero(+)	YES	NO
**HC_COV_78**	M	66	Sero(+)	YES	NO
**p004**	M	64	Sero(+)	YES	NO
**p008**	M	29	Sero(+)	YES	NO
**p018**	F	29	Sero(+)	YES	NO
**p031**	F	42	Sero(+)	YES	NO
**p040**	F	26	Sero(+)	YES	NO
**p052**	M	36	Sero(+)	YES	NO
**p054**	M	62	Sero(+)	YES	NO
**p059**	M	30	Sero(+)	YES	NO
**p061**	F	26	Sero(+)	YES	NO
**p062**	F	36	Sero(+)	YES	NO
**p063**	F	67	Sero(+)	YES	NO
**p078**	M	38	Sero(+)	YES	NO
**p081**	M	35	Sero(+)	YES	NO
**p086**	M	70	Sero(+)	YES	NO
**p111**	M	47	Sero(+)	YES	NO
**p126**	F	44	Sero(+)	YES	NO
**p137**	M	26	Sero(+)	YES	NO
**p156**	M	32	Sero(+)	YES	NO
**p094**	F	47	Mild	YES	YES
**p104**	F	47	Mild	YES	YES
**p105**	M	71	Mild	YES	YES
**p106**	F	37	Mild	YES	YES
**p109**	F	71	Mild	YES	YES
**p112**	F	70	Mild	YES	YES
**p001**	M	81	Moderate	YES	YES
**p043**	M	34	Moderate	YES	YES
**p047**	M	56	Moderate	YES	YES
**p048**	F	57	Moderate	YES	YES
**p058**	F	53	Moderate	YES	YES
**p068**	M	59	Moderate	YES	YES
**p074**	M	40	Moderate	YES	YES
**p075**	M	78	Moderate	YES	YES
**p080**	M	65	Moderate	YES	YES
**p084**	M	28	Moderate	YES	YES
**p088**	F	88	Moderate	YES	YES
**p090**	F	39	Moderate	YES	YES
**p092**	M	84	Moderate	YES	YES
**p099**	M	82	Moderate	YES	YES
**p113**	F	57	Moderate	YES	YES
**p117**	M	68	Moderate	YES	YES
**p016**	M	61	Severe	YES	YES
**p038**	M	56	Severe	YES	YES
**p044**	M	61	Severe	YES	YES
**p046**	F	34	Severe	YES	YES
**p051**	F	66	Severe	YES	YES
**p053**	M	54	Severe	YES	YES
**p073**	M	56	Severe	YES	YES
**p093**	M	84	Severe	YES	YES
**p097**	M	70	Severe	YES	YES
**p108**	M	65	Severe	YES	YES

Table S1. Clinical cohort. Demographics of individual donors. Severity was based on the WHO ordinal scale. "Sero(-)" denotes no evidence of SARS-CoV-2 infection (PCR and/or serological). "Sero(+)" denotes convalescent individuals with evidence of prior SARS-CoV-2 infection (PCR and/or serological). "Mild" denotes individuals with evidence of SARS-CoV-2 infection (PCR) and mild symptoms not requiring hospitalisation. "Moderate" denotes individuals with evidence of SARS-CoV-2 infection (PCR) and symptoms requiring hospitalisation but not necessitating high-flow supplementary oxygen. "Severe" denotes individuals with evidence of SARS-CoV-2 infection (PCR) and symptoms requiring hospitalisation, high-flow supplementary oxygen, intubation and/or organ support as well as individuals who died of COVID-19.

Cell Line	Replicates	Mean Accuracy (S.E.M.)
HUT-78	6	99.5% (0.056%)
Jurkat	174	99.4% (0.067%)

Table S2. ImmunoPETE accuracy. Replicate libraries were produced from clonal T cell lines and accuracy assessed by the percentage of templates recovered with identical nucleotide CDR3s.

IGH Clone ID	Number donors detected	IGH Cluster ID	In CoV-AbDab Database?	Associated with neutralisation?	Target Protein + Epitope	Sources
IGHV4-59_CARGFDYW_IGHJ4	17	1	Yes		S; S2	Li et al., 2021 (doi: 10.1016/j.cell.2021.06.021)
IGHV4-59_CTRGFDYW_IGHJ4	2	1				
IGHV3-30_CAKDYGDYGGLDYW_IGHJ4	1	2	Yes		S; S1 non-RBD	Ehling et al., 2021 (doi: 10.1101/2021.02.12.430940)
IGHV3-30_CAKDYGDYGGFDYW_IGHJ4	1	2				
IGHV3-66_CARDYGDYYFDYW_IGHJ4	3	3	Yes		S; RBD	Chinese Patent (https://patents.google.com/patent/CN111909261A/en)
IGHV3-66_CARAYGDYYFDYW_IGHJ4	1	3				
IGHV3-7_CARVGSSSWYFDYW_IGHJ4	1	4	Yes		S; S2	Chen et al., 2021 (doi: 10.1101/2021.05.02.442326)
IGHV3-7_CARVGSSSWFFDYW_IGHJ4	2	4				
IGHV1-24_CATGPAIAAAATGWFDPW_IGHJ5	1	5	Yes	Yes	S; NTD	Noy-Porat et al., 2021 (doi: 10.1016/j.isci.2021.102479)
IGHV1-24_CATGPGIAEAATGWFDPW_IGHJ5	1	5				
IGHV3-53_CARDLDYYGMDVW_IGHJ6	4	6	Yes	Yes	S; RBD	Wang et al., 2021 (doi: 10.1038/s41586-021-03696-9)
IGHV3-53_CARDLVDYGMDVW_IGHJ6	1	6				
IGHV3-53_CARDLYYYGMDVW_IGHJ6	3	6	Yes	Yes	S; RBD	United States Patent (https://patentimages.storage.googleapis.com/0c/85/52/d9a878e045273a/US10787501.pdf)
IGHV3-53_CARDLVYYGMDVW_IGHJ6	2	6				
IGHV3-53_CARDLAYYGMDVW_IGHJ6	1	6				
IGHV3-53_CARDYGDYYFDYW_IGHJ4	1	7	Yes	Yes	S; RBD	Li et al., 2021 (doi: 10.1016/j.cell.2021.06.021)
IGHV3-53_CARDYGDFYFDYW_IGHJ4	1	7	Yes		S; RBD	Li et al., 2021 (doi: 10.1016/j.cell.2021.06.021)
IGHV3-53_CARDHGGYYFDYW_IGHJ4	1	7				
IGHV3-66_CARDLYYYGMDVW_IGHJ6	2	8	Yes	Yes	S; RBD	Robbiani et al., 2020 (doi: 10.1038/s41586-020-2456-9)
IGHV3-66_CARDLNYYGMDVW_IGHJ6	1	8	Yes		S; RBD	Li et al., 2021 (doi: 10.1016/j.cell.2021.06.021)
IGHV3-66_CARDLVYYGMDVW_IGHJ6	1	8				
IGHV1-58_CAAPNCNSTTCHDGFDIW_IGHJ3	1	NA	Yes		S; RBD	Han et al., 2020 (doi: 10.1101/2020.08.19.253369) and Chinese Patent (https://patents.google.com/patent/CN111909260A/en)
IGHV3-66_CARDLVVYGMDVW_IGHJ6	1	NA	Yes	Yes	S; probably RBD	Cao et al., 2020 (doi: 10.1016/j.cell.2020.05.025)
IGHV3-53_CARDLGPYGMDVW_IGHJ6	1	NA	Yes	Yes	S; RBD	Du et al., 2020 (doi: 10.1016/j.cell.2020.09.035)
IGHV1-58_CAAPYCSGGSCSDAFDIW_IGHJ3	2	NA	Yes	Yes	S; RBD	Robbiani et al., 2020 (doi: 10.1038/s41586-020-2456-9)
IGHV3-53_CAREVYGMDVW_IGHJ6	1	NA	Yes	Yes	S; RBD	Wang et al., 2021 (doi: 10.1038/s41586-021-03696-9)
IGHV3-30_CAKSSGSYYYYYYGMDVW_IGHJ6	1	NA	Yes	Yes (weak)	S; RBD	Rogers et al., 2020 (doi: 10.1126/science.abc7520)
IGHV3-53_CARDFGDFYFDYW_IGHJ4	1	NA	Yes	Yes	S; RBD	Rogers et al., 2020 (doi: 10.1126/science.abc7520) and Yuan et al., 2020 (doi: 10.1126/science.abd2321)
IGHV1-18_CARDGELLGWFDPW_IGHJ5	1	NA	Yes	Yes	S; RBD	Kreer et al., 2020 (doi: 10.1016/j.cell.2020.06.044)
IGHV3-23_CAKEIAVAGCFDYW_IGHJ4	1	NA	Yes		S; non-RBD	Brouwer et al., 2020 (doi: 10.1126/science.abc5902)
IGHV4-59_CARGPAATYYYYMDVW_IGHJ6	1	NA	Yes		S; non-RBD	Brouwer et al., 2020 (doi: 10.1126/science.abc5902)
IGHV1-2_CARLDYW_IGHJ4	1	NA	Yes		S; Unk	Mullen et al., 2021 (doi: 10.1101/2021.05.31.446421)
IGHV3-15_CTTHSSPDYW_IGHJ4	1	NA	Yes		S; Unk	Mullen et al., 2021 (doi: 10.1101/2021.05.31.446421)

Table S3. Public, SARS-CoV-2 reactive IGH sequences. We found 23 unique exact IGH sequence matches in our dataset with sequences published in the the CoV-AbDab database (matched on V gene, CDR3 and J gene), as well as 10 additional sequences closely related to these 23 sequences by change-o clustering. Columns denote, from left to right, the number of donors each sequence was identified in, presence in change-o clusters indicated by cluster ID, presence as an exact match in the CoV-AbDab database, association with neutralising activity, target protein/epitope and the primary source identifying SARS-CoV- 2 reactivity.

	SARS-CoV-2 Exposed Donors	Sero(-) Donors	Total
CoV-AbDab Sequence Present	**27**	**3**	**30**
CoV-AbDab Sequence Absent	**25**	**40**	**65**
Total	**52**	**43**	
Fisher's exact test (one sided)		**p<0.0001**	

Table S4. SARS-CoV-2 reactive IGH sequences significantly enriched in exposed individuals. Contingency table showing the distribution SARS-CoV-2 reactive IGHV-CDR3-IGHJ sequences identified in CoV-AbDab amongst SARS-CoV-2 exposed and sero(-) individuals.

	# unique TCRs for clustering	# of raw clusters	# clusters enriched for SARS-CoV-2 exposed individuals	# TCRs in clusters enriched for SARS-CoV-2 exposed individuals	# clusters enriched for sero(-) individuals
**CD4+**	2,091,619	11,269,019	2,993	21,869	1
**CD4-**	897,211	1,122,161	511	3,458	0

Table S5. TCRs clustered. Note, the numbers of clusters exceed the numbers of TCRs as each TCR can belong to multiple clusters. Significance established by one-sided Fisher's exact test $p<0.05$.

Supplementary Materials and Methods

Study Design and Human Subjects and Samples

Peripheral blood draws were obtained from patients and healthy control individuals as part of the COVID-IP study between 14th April 2020 and 21st July 2020 as previously described (1). The active COVID-19 cohort (n=32) was composed of adult patients treated at Guy's and St Thomas' Hospitals (London, UK) with PCR proven SARS-CoV-2 infection on nasopharyngeal swab. Baseline peripheral blood samples in the active COVID-19 cohort were taken as soon as possible after a positive PCR result for SARS-CoV-2 (median=4 days, IQR=2-9.75 days). A subset of patients in the active COVID-19 cohort (13/32) had additional peripheral blood draws ~3 days post baseline sampling and also variably at later timepoints. Patients in the active COVID-19 cohort were classified as having "mild" (not requiring supplemental oxygen), "moderate" (requiring less than 40% supplemental oxygen) or "severe" (requiring ≥40% supplemental oxygen and/or ≥ level 2 critical care) disease based on the WHO ordinal scale. Healthy control samples were obtained from 63 individuals drawn largely from a pool of healthcare workers and research scientists working at King's College London and Guy's and St. Thomas' Hospitals. A subset of these individuals (9/63) also had additional peripheral blood draws at later timepoints. All individuals had SARS-CoV-2 antibody titres determined by ELISA as previously described (1). Healthy control individuals were thus further categorised on the basis of SARS-CoV-2 serology as sero(+) (n=20) or sero(-) (n=43). A few sero(+) donors (3/20) were symptomatic previously and had previous PCR proven SARS-CoV-2 infection. The majority of sero(+) donors (17/20) had presumed asymptomatic infection without PCR evidence. A total of 125 peripheral blood samples were collected (including longitudinal timepoints) consisting of 52 active COVID-19 samples, 26 sero(+) samples and 47 sero(-) control samples. "Early" timepoint samples were collected within 14 days of symptom onset whilst "late" timepoint samples were collected after 14 days of symptom onset. "Unknown" timepoint samples were collected from asymptomatic individuals. There was a single "late" timepoint sample from a sero(+) individual aged <50 which was analysed with aged <50 unknown timepoint samples to increase statistical power.

Patient and healthy control samples were collected, with written informed consent, under the ethics approval of the Infectious Diseases Biobank of King's College London with reference

numbers COV-250320 and MJ1-031218b respectively. Both approvals were granted under the terms of the Infectious Disease Biobank of King's College London ethics permission (reference 19/SC/0232) granted by the South Central Hampshire B Research Ethics Committee in 2019. We complied with all relevant ethical regulations.

PCR for SARS-CoV-2 Detection

Nasopharyngeal swabs were collected from patients suspected to have COVID-19 or for routine screening from those regularly attending or admitted to hospital for other reasons. Nucleic acid extraction and PCR were performed as previously described for the COVID-IP study using the AusDiagnostics two-step multiplexed-tandem PCR assay (Coronavirus Typing Eight-well Panel; cat. no. 2061901) or AusDiagnostics SARS-CoV-2, Influenza, RSV (eight-well) Panel (cat. no. 80081) (1).

Sample Processing and PBMC Isolation

Full methodology for sample processing is described in detail in the COVID-IP study (1). Briefly, whole blood samples were processed in Biosafety Level 3 containment conditions as per local code of practice approved by King's College London. Whole blood was diluted 1:1 with PBS and peripheral blood mononuclear cells (PBMCs) obtained by Ficoll density gradient separation. Approximately 20% of the PBMC fraction from each blood draw was used for downstream magnetic-activated cell sorting (MACS) and DNA extraction for antigen receptor sequencing and HLA typing as described below.

SARS-CoV-2 Serology

SARS-CoV-2 serology was determined by ELISA using diluted plasma from Ficoll density gradient separation as previously described in the COVID-IP study (1). Titers were normalized using a min/max normalization to compare samples across batches within the COVID-IP study. Cut-offs were determined based on data distribution with respect to healthy controls and values >0.15 were considered as positive. Sero(+) samples were positive for anti-spike and/or anti-RBD. Negative samples or samples positive only for anti-nucleoprotein were classified as sero(-).

MACS Sorting of PBMCs

PBMCs aliquots (~2-20 million cells) from whole blood isolation were MACS sorted with CD4 Microbeads (Miltenyi) yielding a highly pure $CD4^+$ T cell fraction and a $CD4^+$ depleted PBMC fraction containing $CD8^+$ αβ T cells, γδ T cells and B cells. Sorting was carried out as per manufacturer's instructions with minor modifications to optimize the purity of both $CD4^+$ and $CD4^-$ fractions. Briefly, PBMCs were counted, washed and resuspended in 80µl of sterile MACS buffer (PBS, 2% foetal bovine serum, 2mM EDTA) for every 5 x 10^6 cells up to a maximum of 320ml for 2 x 10^7 cells. PBMCs in suspension were incubated with 20µl of CD4 Microbeads for every 5 x 10^6 cells for 15 minutes at 4°C and then washed with MACS buffer before resuspending in 1ml of MACS buffer. This suspension was then applied to an MS column (Miltenyi) in sequential 500µl aliquots and flowthrough collected as the $CD4^-$ fraction. Columns were washed three times with 500µl of MACS buffer and flowthrough also collected as the $CD4^-$ fraction. Columns were then forcibly flushed with 1ml of MACS buffer to harvest the $CD4^+$ fraction. Care was taken to keep reagents and cells at 4°C to minimise activation and non-specific labelling. The cell suspensions for both fractions were washed, counted and resuspended in approximately 20µl of MACS buffer. A small aliquot (~2µl) from each sample was taken for flow cytometry to assess relative frequencies of T and B cell subsets (see flow cytometry below). The remainder (~18µl) was lysed in RLT plus buffer + 10µl/ml of 2-mercaptoethanol (Qiagen) and frozen at -80°C for subsequent nucleic acid extraction using the Allprep DNA/RNA mini kit (Qiagen, see DNA extraction below).

Flow Cytometry

MACS sorted cell fractions were stained for 15 mins at 4°C in 50µl of MACS buffer and antibody mastermix [anti-CD3 APC (Biolegend), anti-CD4 PerCP/Cy5.5 (Biolegend), anti-CD8 APC/Cy7 (Biolegend), anti-TCRγδ PE/Cy7 (Beckman Coulter) and anti-CD19 FITC (Biolegend), all at 1:100 dilution]. Cells were then washed twice with MACS buffer and resuspended in fix buffer for 15 minutes (BD CellFIX). Fixed samples were acquired on a BD LSR Fortessa flow cytometer and results analyzed using FlowJo (Treestar/BD). $CD3^+$/$TCR\gamma\delta^-$ cells were considered to be αβ T cells.

Correlations with immune parameters from the COVID-IP study

Many of the donors included in this study also had in-depth immunophenotyping as part of the COVID-IP study(1). Figures 1C, 1D, 1E, 2D, 2E, 2H, 4D present correlations between repertoire metrics and immune parameters measured by the COVID-IP study in the same donors. Methodology and source data are available online at https://www.immunophenotype.org/index.php/covid-ip/ as well as from the primary manscript (1).

DNA Extraction, Quantification and Quality Assessment

DNA was extracted from cell lysates using the AllPrep DNA/RNA Mini Kit (Qiagen) as per manufacturer's instructions with minor modifications as detailed below. Briefly, lysates were homogenised using QIAshredder columns (Qiagen) as per manufacturer's instructions. Homogenised lysates were applied to AllPrep DNA spin columns and washed successively with Buffer AW1 and AW2 as per manufacturer's instructions. Fully washed columns were incubated at room temperature for 5 minutes with 50ml of nuclease free water pre-heated to 70°C and DNA eluted by centrifugation at ≥8000g for one minute. The eluate was reapplied to the column for a second elution to maximise DNA yields. Quantification was performed using the Qubit dsDNA HS Assay kit (Thermo Fisher) as per manufacturer's instructions. DNA quality was measured by absorbance ratios at 260nm/280nm and 260nm/230nm using a Nanodrop spectrophotometer (Thermo Fisher).

HLA Typing

Approximately 500ng of gDNA or 500µl of whole blood from each donor was sent to Viapath Analytics (London, UK) for HLA typing using the Lifecodes Rapid SSO HLA Typing Kits.

Generating Antigen Receptor Libraries and NGS

A next generation sequencing (NGS) library of the adaptive immune repertoire of each sample was generated using the Immuno-PETE method. Immuno-PETE is a modification of Roche Sequencing Solutions PETE method [patent US10731212B2]. In this adaptation, the primer extension based targeted gene enrichment assay was designed to specifically enrich and amplify human T-cell receptor (TCR) and B-cell receptor (BCR/Ig) loci from genomic DNA. It is

optimized for the human TCRβ (TRB), TCRδ (TRD) and Ig heavy (IGH) chain receptors and uses Illumina NextSeq platforms for sequencing.

From gDNA, an initial single V gene-based primer extension was performed. V gene oligos contain a unique molecular identifier (UMI) sequence as well as a universal amplification sequence at the 3' end. Following V gene-based primer extension, treatment with Thermolabile Exonuclease I (New England Biolabs) and a subsequent bead-based purification (KAPA HyperPure, Roche) removed remaining oligos. Thereafter, a master-mix of a pool of J gene oligos with an i7-primer was added to purified V gene-primed templates for J gene primer extension with a 10-cycle target amplification. This was followed by Illumina library amplification using i7/i5-sequencing primers with dual unique indexes. All primer extensions and amplifications were performed using the KAPA Long Range HotStart Ready Mix (Roche). Resulting libraries were purified using KAPA HyperPure beads (Roche), before quantification with the Qubit dsDNA HS Assay kit (Thermo Fisher) and fragment analysis on a TapeStation (Agilent). Libraries were pooled in equal mass to create a library pool before another round of quantification and fragment analysis prior to sequencing using the Illumina NextSeq 500/550 High Output Kit v2.5 (300 cycle).

Data Processing

A Roche in-house bioinformatics pipeline was used to process sequencing reads. Immuno-PETE leverages UMIs to enable counting of T and B cells at single molecule resolution. Read pairs were quality filtered and trimmed of adapter and primer sequences. V and J genes were identified by a Smith-Waterman alignment against the HGNC reference gene annotations. Using a minimum alignment accuracy of 90%, the method typically achieves a 37% on-target rate, which is defined as the percent of paired-end reads that span a putative VDJ recombinant (contains both the V and J-genes) over the total number of paired-end reads. After converting the aligned nucleotide sequences into amino acid (AA) sequences, the CDR3 regions were predicted for all V-J pairs using known V/D and D/J junction identifiers, and characterized as functional and non-functional rearrangements. UMI and CDR3 sequences were clustered together, defining UMI-families (all reads originating from a single molecule). The UMI-family sizes, or number of reads supporting a UMI-family, consistently displayed a log-normal distribution indicating minimal amplification bias in the assay. In order to suppress

random errors that occur from sequencing or PCR amplification, consensus sequences were derived for all UMI families with two or more reads. Quality score filters were also used to filter low-quality consensus reads, resulting in high quality CDR3 sequence predictions and accurate cell counts. While these common methods to reduce noise in next generation sequencing data help to minimize sequencing and PCR noise, due to the hypervariability of immune cell receptor sequences, we also used D99 to differentiate between putative CDR3 signatures from remaining artefactual singletons. These combined strategies provide unbiased and quantitative TCR and BCR repertoire information with next-generation sequencing analysis. Functional rearrangements were defined by V gene, CDR3 AA sequences, and J gene combinations excluding rearrangements containing pseudogenes. “Hybrid” rearrangements (e.g. TRBV-CDR3-IGHJ, TRDV-CDR3-TRBJ, etc.) were also excluded. All analyses were conducted using functional rearrangements (excluding hybrids) based on CDR3 AA sequences unless otherwise specified. Code is provided at [github location].

Diversity/Clonality Analyses

The frequencies of matching V genes + CDR3 AA + J genes were used to calculate entropy/dominance metrics across all 4 cell types:$CD4^+$ αβ T cells ($CD4^+$ TRB), $CD8^+$ αβ T cells ($CD4^-$ TRB), γδ T cells(TRD) and B cells(IGH). Metrics were normalized to account for differences in the total number of cells per sample and calculated as detailed below. Treemaps were generated using the MacroFocus Treemap programme (https://www.treemap.com).

$$R \ = number\ of\ clones\ (each\ TCR\ may\ be\ present\ in\ several\ cells)$$

$$N \ = sum\ of\ cell\ counts\ across\ all\ clones\ = \sum_{i=1}^{R} n_i$$

$$Frequency\ of\ each\ clone\ p : \sum_{i=1}^{R} p_i = 1$$

$$Shannon's\ entropy = \frac{-\sum_{i=1}^{R} p_i log_2(p_i)}{log_2(R)}$$

$$Simpson's\ dominance = \sum_{i=1}^{R} p_i^2$$

$$D_{50} = \frac{C}{R}\ where\ clones\ are\ sorted\ decreasing\ and\ C\ is\ smallest\ possible\ C, where \sum_{i=1}^{C} n_i \geq \frac{N}{2}$$

IGH Clustering

We used the DefineClones.py script in the change-o toolbox (version 1.0.0 2020.05.06) to cluster IGH CDR3 clones. Diversity measures were also calculated using IGH clusters, accounting for the impact of somatic hypermutation (SHM) on the estimate of B-cell diversity.

TRB Sub-sampling

Each sample was sub-sampled to 1200 ($CD8^+$) or 2400 ($CD4^+$) cells by drawing TCRs (defined as V gene + CDR3 AA + J gene) with replacement with probability equal to their presence in the starting sample. Samples with less than 1200 ($CD8^+$) or 2400 ($CD4^+$) cells were not included. Medians of metrics computed from 100 resamples (entropy/dominance metrics) were reported as "sub-sampled" values.

TRB Clustering

For each HLA gene, TCR sequences were collated from across all individuals from the study without sub-sampling ($CD4^+$ for HLA class II and $CD8^+$ for HLA class I), irrespective of their SARS-CoV-2 exposure status. Only HLA backgrounds with at least 8 individuals and at least 4 SARS-CoV-2 exposed individuals were included in subsequent analyses. Unique TCRs (TRBV-CDR3-J) were clustered within each HLA background with GLIPH2 (http://50.255.35.37:8080/, executables from October 2020), ignoring the first and the last 3 amino acids of the CDR3 and with requirements for CDR3 length to be at least 8 amino acids, k-mers of size 2-4, allowing only BLOSUM62 positive amino acid replacements and with all other settings as default. Raw clusters were tested with a one-sided Fisher exact test for overrepresentation of SARS-CoV-2 exposed individuals (cut-off $p<0.05$). For the reciprocal comparison, we performed the same analysis, but conditioning on HLA presence in sero(-) individuals and requiring overrepresentation of this set of individuals in the clusters. Because of the heavily nested and intercorrelating nature of TCR clusters and related multiple hypotheses, no standard multiple testing correction method was applicable. However, if such clustering enrichments were to arise by chance, we would expect to see significant clusters comprising only sero(-) individuals, which was not the case.

Statistical Analysis

Statistical tests were conducted using Prism 9 (GraphPad), JMP Pro 15 and R version 4.0.3 + RStudio with CRAN available packages. Statistical tests used are specified in accompanying figure legends. All tests were two-sided unless otherwise specified.

References

1. A. G. Laing, *et al.*, A dynamic COVID-19 immune signature includes associations with poor prognosis. *Nature Medicine* **26**, 1623–1635 (2020).

Key Reagents/Resources Table

REAGENT or RESOURCE	SOURCE	IDENTIFIER
**Antibodies**		
anti-CD3 APC (Clone:OKT3, Lot:B222493)	Biolegend	Cat# 317318; RRID:AB_1937212
anti-CD4 PerCP/Cyanine5.5 (Clone:OKT4, Lot:B278611)	Biolegend	Cat# 317428; RRID:AB_1186122
anti-CD8 APC/Cy7 (Clone:HIT8a, Lot: B211497)	Biolegend	Cat# 300926; RRID:AB_10613636
anti-CD19 FITC (Clone:HIB19, Lot:B274550)	Biolegend	Cat# 302206; RRID:AB_314236
anti-CD45 Pacific Blue (Clone:HI30, Lot:B263330)	Biolegend	Cat# 304022; RRID:AB_493655
anti-TCRγδ PE/Cy7 (Clone:IMMU510, Lot: 200044)	Beckman Coulter	Cat# B10247
**Biological Samples**		
PBMCs from Human Subjects	COVID-IP Study (16)	COVID-IP Study (16)
**Chemicals, Peptides, and Recombinant Proteins**		
AllPrep DNA/RNA Mini Kit (inc. RLT Plus Buffer)	Qiagen	Cat# 80204
BD CellFIX (10x concentrate)	BD Biosciences	Cat# 340181
2-Mercaptoethanol >99%	BDH Laboratory Supplies	Cat# 436024C
QIAshredder	Qiagen	Cat# 79654
**Critical Commercial Assays and Reagents**		
CD4 MicroBeads, human	Miltenyi Biotec	Cat# 130-045-101
High Sensitivity D5000 Reagents	Agilent	Cat# 5067-5593
High Sensitivity D5000 Screentape	Agilent	Cat# 5067-5592
KAPA HyperPure Beads	Roche	Cat# KK8002
KAPA Long Range HotStart Ready Mix (2X) with Dye	Roche	Cat# KK3602
MS Columns	Miltenyi Biotec	Cat# 130-042-201
Thermolabile Exonuclease I	New England Biolabs	Cat# M0568L
Qubit dsDNA HS Assay Kit	Thermo Fisher Scientific	Cat# Q32851
**Experimental Models: Cell Lines**		
P116 Jurkat Cell Line	Francis Crick Institute	ATCC Cat# CRL-2676; RRID:CVCL_6429
**Software and Algorithms**		
FlowJo	Treestar/BD	https://www.flowjo.com
GLIPH2	Huang et al., 2020	http://50.255.35.37:8080/
JMP Pro 15		https://www.jmp.com/en_gb/home.html
MacroFocus Treemap	MacroFocus	https://www.treemap.com
Prism 9	GraphPad	https://www.graphpad.com
R version 4.0.3 + RStudio		https://www.r-project.org
